# Antigen specificity and cross-reactivity drive functionally diverse anti–*Aspergillus fumigatus* T cell responses in cystic fibrosis

**DOI:** 10.1172/JCI161593

**Published:** 2023-03-01

**Authors:** Carsten Schwarz, Patience Eschenhagen, Henrijette Schmidt, Thordis Hohnstein, Christina Iwert, Claudia Grehn, Jobst Roehmel, Eva Steinke, Mirjam Stahl, Laura Lozza, Ekaterina Tikhonova, Elisa Rosati, Ulrik Stervbo, Nina Babel, Jochen G. Mainz, Hilmar Wisplinghoff, Frank Ebel, Lei-Jie Jia, Matthew G. Blango, Peter Hortschansky, Sascha Brunke, Bernhard Hube, Axel A. Brakhage, Olaf Kniemeyer, Alexander Scheffold, Petra Bacher

**Affiliations:** 1 Klinikum Westbrandenburg, Campus Potsdam, Cystic Fibrosis Section, Potsdam, Germany.; 2 Institute of Clinical Molecular Biology, Christian-Albrecht University of Kiel, Kiel, Germany.; 3 Institute of Immunology, Christian-Albrecht University of Kiel and UKSH Schleswig-Holstein, Kiel, Germany.; 4 Department of Microbiology, Infectious Diseases and Immunology, Charité – Universitätsmedizin Berlin, Berlin, Germany.; 5 Berlin Institute of Health at Charité – Universitätsmedizin Berlin, Translational Immunology, Berlin, Germany.; 6 Berlin Institute of Health at Charité – Universitätsmedizin Berlin, Berlin, Germany.; 7 Department of Pediatric Respiratory Medicine, Immunology and Critical Care Medicine and Cystic Fibrosis Center, Charité – Universitätsmedizin Berlin, corporate member of Freie Universität Berlin and Humboldt – Universität zu Berlin, Berlin, Germany.; 8 German Center for Lung Research (DZL), associated partner site, Berlin, Germany.; 9 Cell Biology Laboratory, Precision for Medicine GmbH, Berlin, Germany.; 10 Center for Translational Medicine and Immune Diagnostics Laboratory, Marien Hospital Herne, University Hospital of the Ruhr University Bochum, Herne, Germany.; 11 Brandenburg Medical School/Medizinische Hochschule Brandenburg (MHB), University, Pediatric Pulmonology/Cystic Fibrosis, Klinikum Westbrandenburg, Brandenburg an der Havel, Germany.; 12 Labor Dr. Wisplinghoff, Cologne, Germany.; 13 Institute for Virology and Microbiology, Witten/Herdecke University, Witten, Germany.; 14 Institute for Infectious Diseases and Zoonoses, LMU, Munich, Germany.; 15 Department of Molecular and Applied Microbiology, Leibniz Institute for Natural Product Research and Infection Biology (Leibniz-HKI), Jena, Germany.; 16 Department of Microbial Pathogenicity Mechanisms, Leibniz Institute for Natural Product Research and Infection Biology (Leibniz-HKI), Jena, Germany.; 17 Institute of Microbiology, Friedrich Schiller University, Jena, Germany.

**Keywords:** Immunology, Pulmonology, Adaptive immunity, Fungal infections

## Abstract

**BACKGROUND:**

The fungus *Aspergillus fumigatus* causes a variety of clinical phenotypes in patients with cystic fibrosis (pwCF). Th cells orchestrate immune responses against fungi*,* but the types of *A*. *fumigatus*–specific Th cells in pwCF and their contribution to protective immunity or inflammation remain poorly characterized.

**METHODS:**

We used antigen-reactive T cell enrichment (ARTE) to investigate fungus-reactive Th cells in peripheral blood of pwCF and healthy controls.

**RESULTS:**

We show that clonally expanded, high-avidity *A*. *fumigatus*–specific effector Th cells, which were absent in healthy donors, developed in pwCF. Individual patients were characterized by distinct Th1-, Th2-, or Th17-dominated responses that remained stable over several years. These different Th subsets target different *A*. *fumigatus* proteins, indicating that differential antigen uptake and presentation directs Th cell subset development. Patients with allergic bronchopulmonary aspergillosis (ABPA) are characterized by high frequencies of Th2 cells that cross-recognize various filamentous fungi.

**CONCLUSION:**

Our data highlight the development of heterogenous Th responses targeting different protein fractions of a single fungal pathogen and identify the development of multispecies cross-reactive Th2 cells as a potential risk factor for ABPA.

**FUNDING:**

German Research Foundation (DFG), under Germany’s Excellence Strategy (EXC 2167-390884018 “Precision Medicine in Chronic Inflammation” and EXC 2051-390713860 “Balance of the Microverse”); Oskar Helene Heim Stiftung; Christiane Herzog Stiftung; Mukoviszidose Institut gGmb; German Cystic Fibrosis Association Mukoviszidose e.V; German Federal Ministry of Education and Science (BMBF) InfectControl 2020 Projects AnDiPath (BMBF 03ZZ0838A+B).

## Graphical abstract

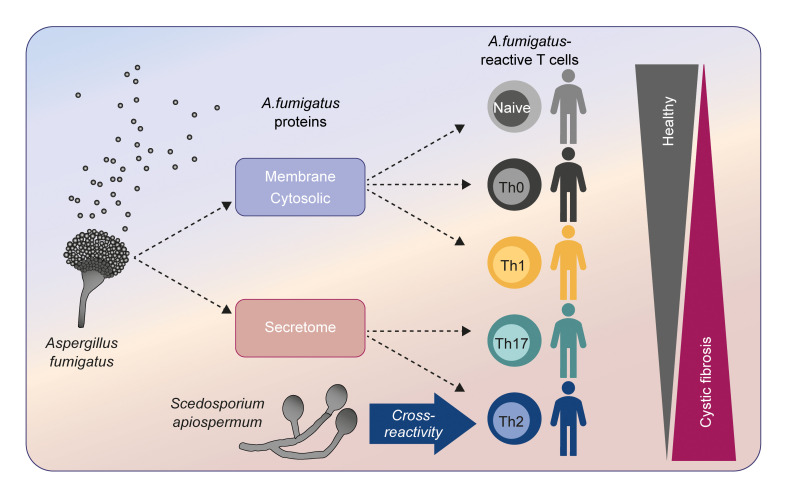

## Introduction

Humans are constantly exposed to ubiquitous airborne fungi such as *Aspergillus fumigatus*. Despite this continuous challenge, healthy individuals rarely develop *Aspergillus*-associated diseases. This is likely due to rapid clearing of inhaled fungal spores by mucociliary clearance and coughing (1), as well as innate immune cells (2). We previously showed that healthy individuals also develop strong Treg responses against *A*. *fumigatus* that prevent inappropriate immune reactions such as Th2-mediated allergies (3, 4).

In contrast, patients with cystic fibrosis (pwCF) experience disease exacerbations due to recurrent and chronic infections of the lung (5, 6). *A*. *fumigatus* is the most common filamentous fungus found in up to 57% of respiratory secretions from adult pwCF (7, 8). *A*. *fumigatus* rarely causes invasive or systemic infections in pwCF, but these patients often develop allergic hypersensitivity against the fungus. Allergic reactions to *A*. *fumigatus* form a spectrum of different diseases ranging from IgE-mediated sensitization to the severe allergic complication allergic bronchopulmonary aspergillosis (ABPA) (9, 10). Rare *Aspergillus*-associated pathologies include bronchitis, aspergilloma, and pneumonia (11–15). These different diseases are difficult to diagnose, and the precise contribution of *A*. *fumigatus* is not clear.

The wide spectrum of clinical manifestations caused by *A*. *fumigatus* suggests different fungus-host interactions in pwCF. However, the types of anti–*A*. *fumigatus* immune responses and the extent to which they contribute to protection or pathology are poorly understood. This knowledge would be essential for developing disease- and patient-specific clinical interventions. CD4^+^ Th cells are the central organizers of adaptive immunity against fungi (16). Th cell responses can display considerable heterogeneity, reflecting different types of immune reactions ranging from tolerogenic, protective, nonprotective, to even pathogenic responses, such as hypersensitivity. Therefore, the characterization of Th cells specific for a defined pathogen can provide important information about the actual host-pathogen interaction status of an individual.

Several studies have found low frequencies of predominantly IFN-γ–producing T cells in response to *A*. *fumigatus* in healthy individuals (reviewed in ref. 17). However, many of these studies used technologies to detect antigen-specific T cells that are limited in terms of sensitivity and provided no further characterization regarding the quality of the anti–*A*. *fumigatus* T cell response. Using the sensitive antigen-reactive T cell enrichment (ARTE) technology (3, 4, 18–20), we previously identified *A*. *fumigatus*–reactive CD4^+^ T cells in essentially all healthy individuals, but a large fraction of these cells maintain a naive phenotype (3, 4, 19). In contrast, the T cell reaction to *A*. *fumigatus* is dominated by memory Tregs, which may actively prevent the activation of conventional naive cells (3, 4). Thus, the CD4^+^ T cell response against *A*. *fumigatus* in healthy individuals mirrors the response to harmless aeroantigens, such as plant pollen or house dust mites (3).

Despite the clinical importance, data on *A*. *fumigatus*–specific T cells in pwCF are rare. Expansion of Th2 cells, which are virtually absent in healthy individuals, has been demonstrated in a subgroup of pwCF (3, 4, 21–25). Furthermore, increased frequencies of *A*. *fumigatus*–reactive Th17 cells are found in the blood and lungs of pwCF (18, 26) and have been associated with allergic lung inflammation in ABPA (18). Reduced Treg frequencies and impaired suppression capacity were identified in pwCF (27), but *A*. *fumigatus*–specific Tregs have not yet been examined. Thus, the functional and clinical importance of different *A*. *fumigatus*–reactive Th cell reaction types and their heterogeneity within patients are poorly understood. A better understanding of anti–*A*. *fumigatus* T cell responses would have great potential for subclassification of individuals, identification of patients at risk, and development of novel and individualized therapeutic approaches.

Here, we show that pwCF, but not healthy donors, harbor functionally distinct effector T cell responses, indicating active fungus-host interactions in CF. The individual Th reactivity pattern remained stable over several years, and different Th subsets target distinct *A*. *fumigatus* proteins. During acute ABPA, Th2 cells exhibiting cross-reactivity to several filamentous fungi expand, highlighting a potential role of other commonly encountered fungal species for the development and/or exacerbation of ABPA. Our results provide a unique example of heterogenous Th cell reactions against a single fungal pathogen, which are directed against different *A*. *fumigatus* protein fractions. These data help to define different *A*. *fumigatus*–associated pathologies in pwCF and to reveal specific therapeutic intervention points.

## Results

### Clonally expanded, high-avidity memory Th cell responses against A. fumigatus in pwCF.

**Figure 1:**
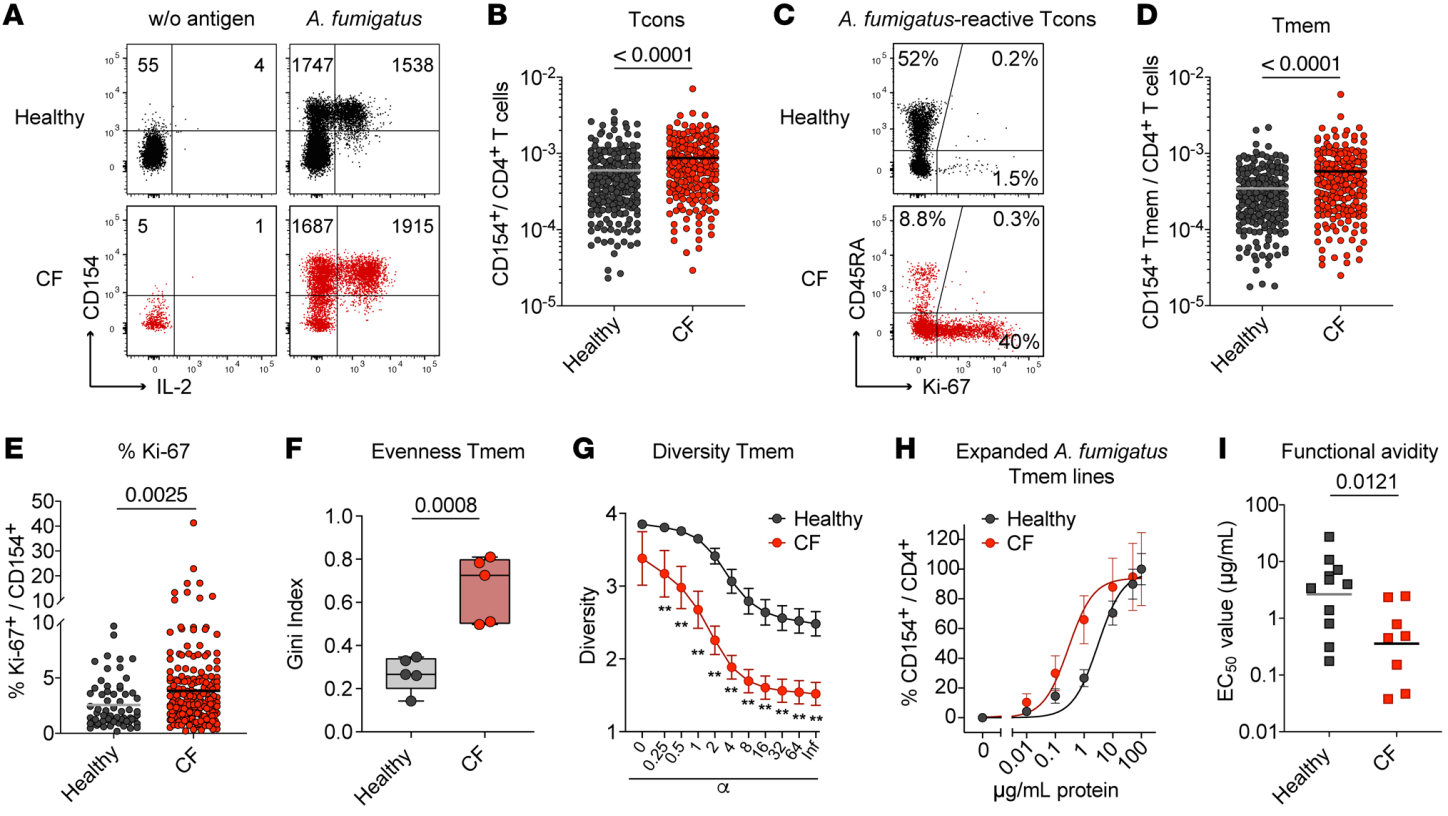
*A*. *fumigatus*–reactive conventional CD4^+^ T cell responses in pwCF. (**A**) Dot plot examples for the ex vivo detection of *A*. *fumigatus*–reactive CD4^+^ T cells by ARTE. PBMCs (1 × 10^7^) were stimulated with *A*. *fumigatus* or left unstimulated. Cell counts before and after magnetic CD154^+^ enrichment are indicated in the plots. (**B**) Frequencies of *A*. *fumigatus*–reactive CD154^+^CD4^+^ T cells in healthy donors (*n* = 220) and pwCF (*n* = 200). (**C**) CD45RA and Ki-67 staining of *A*. *fumigatus*–reactive CD154^+^ T cells. The percentage of marker-positive cells within the CD154^+^ population is indicated. (**D**) Frequencies of *A*. *fumigatus*–reactive CD154^+^CD45RA^–^memory CD4^+^ T cells (Tmem) in healthy donors (*n* = 220) and pwCF (*n* = 200). (**E**) Ki-67 expression of *A*. *fumigatus*–reactive CD154^+^ T cells (healthy individuals, *n* = 65; pwCF, *n* = 200). (**F** and **G**) TCR-β sequence analysis of the top 50 expanded *A*. *fumigatus*–specific T cell clones from healthy individuals and pwCF (*n* = 5 for both). (**F**) Gini index depicting the distribution of TCR sequences (0 is total equality, i.e., all clones have the same proportion; 1 is total inequality, i.e., a population dominated by a single clone). (**G**) Rényi diversity profiles. Values of α = 0, 1, 2, and infinite, correspond to the richness, Shannon diversity, Simpson diversity, and Berger-Parker index, respectively. The sample with the highest value at α = 0 has the highest richness, but the lower value at α = infinite indicates a higher proportion of the most abundant sequence, i.e., lower population diversity. A sample with a profile that is overall higher than the profiles of other samples is therefore more diverse. (**H** and **I**) *A*. *fumigatus*–reactive CD154^+^ Tmem cells from healthy donors (*n* = 11) and pwCF (*n* = 8) were FACS purified, expanded, and restimulated in the presence of autologous FastDCs derived from blood monocytes. (**H**) Dose-response curves of expanded T cell lines, restimulated with decreasing antigen concentrations. (**I**) EC_50_ values were calculated from dose-response curves. Each symbol in **B**, **D–F**, and **I** represents 1 donor; horizontal lines indicate the mean in **B**, **D**, and **E** and the geometric mean in **I**. Box-and-whisker plots display quartiles and range in **F**. Data indicate the mean ± SEM in **G** and **H**. Statistical differences were determined by 2-tailed Mann-Whitney *U* test in **B**, **D**, **E**, and **I**; 2-tailed, unpaired *t* test in **F**; and Kruskal-Wallis test in **G**.

We analyzed the CD4^+^ T cell reaction against *A*. *fumigatus* in 220 healthy individuals and 200 pwCF (see Supplemental Table 1; supplemental material available online with this article; https://doi.org/10.1172/JCI161593DS1 for demographic data) using ARTE technology (3, 4, 18, 20). Patients and controls were enrolled in the study between the years 2013 and 2018. *A*. *fumigatus*–reactive conventional T cells (Tcons) were detected following 7 hours of ex vivo stimulation of PBMCs with *A*. *fumigatus* lysate and subsequent magnetic enrichment of CD154–expressing (CD40L-expressing) CD4^+^ T cells (Figure 1A and Supplemental Figure 1A). This enabled sensitive detection of *A*. *fumigatus–*reactive T cells with a high signal-to-noise ratio (mean, 50; range, 2.5–850) and a mean detection of 2,000 CD154^+^ cells (range, 40–15,333 cells) after subtraction of background events (Supplemental Figure 1, B–D). We confirmed the specificity of the *A*. *fumigatus* lysate–stimulated T cells by restimulation of expanded CD154^+^ T cells (Supplemental Figure 1, E and F) and strongly diminished CD154 induction upon blockade of antigen presentation via anti–MHC-II antibodies (Supplemental Figure 1, G and H) (3, 20). Compared with healthy individuals, pwCF had slightly increased frequencies of *A*. *fumigatus*–reactive Tcons (Figure 1B). To characterize the reactive T cells further, we stained for CD45RA to discriminate between naive and antigen-experienced memory T (Tmem) cells. A substantial fraction of the *A*. *fumigatus*–reactive Tcons from healthy donors had a CD45RA^+^ naive phenotype, confirming previous results (3, 4). In contrast, *A*. *fumigatus*–reactive T cells from pwCF showed an increased proportion of memory cells (Tmem) (Figure 1C). Accordingly, significantly elevated frequencies of *A*. *fumigatus*–reactive Tmem cells were detected in pwCF (Figure 1D). Furthermore, reactive T cells from pwCF displayed increased expression of the proliferation marker Ki-67, indicative of a recent in vivo interaction with fungal antigens (Figure 1, C and E).

We next analyzed the T cell receptor (TCR) repertoire of the *A*. *fumigatus*–reactive Tmem cells. T cells from pwCF showed increased clonal expansions, indicated by a higher Gini index, as a measure of the evenness of a population (Figure 1F). Consistently, *A*. *fumigatus*–reactive Tmem cells from pwCF were less diverse, as seen by a lower Rényi diversity profile (Figure 1G). To analyze functional avidity, we expanded *A*. *fumigatus*–reactive Tmem cells in vitro and rechallenged them with decreasing antigen concentrations in the presence of autologous fast DCs differentiated from monocytes. Tmem cells from pwCF had a 10-fold higher functional avidity compared with *A*. *fumigatus*–reactive Tmem cells from healthy controls (Figure 1, H and I).

Collectively, these data show that clonally expanded, high-avidity memory T cells against *A*. *fumigatus* were present in pwCF, whereas healthy individuals lacked the typical characteristics of such an in vivo antigen–primed Tmem response (4, 18). This suggests that, although all humans are frequently exposed to ubiquitous *A*. *fumigatus* conidia, the development of effector T cell responses probably requires more intense or prolonged fungal exposure or a local barrier defect, as is the case in CF lungs. However, differences in the subset composition or functionality of antigen-presenting cell subsets have been described in pwCF (28), which may also affect the processing of individual proteins or the activation of certain T cell specificities or functional subsets by ARTE.

### A. fumigatus–reactive Tregs are not altered in pwCF.

**Figure 2:**
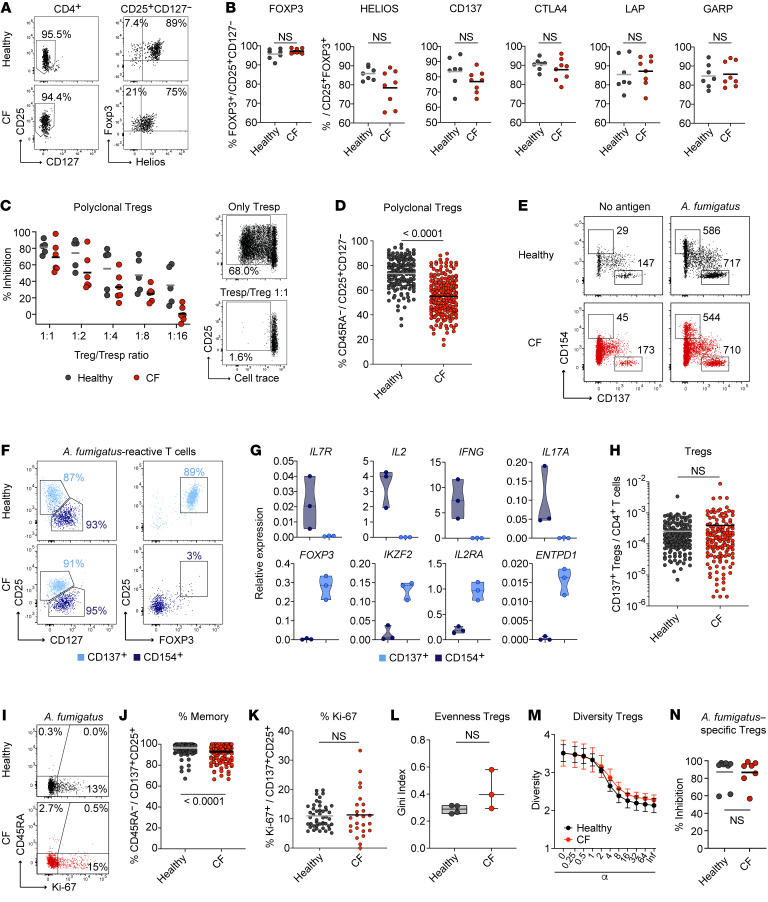
*A*. *fumigatus*–reactive regulatory CD4^+^ T cell responses in pwCF. (**A**) Dot plot examples of purified CD25^+^CD127^–^ Tregs from PBMCs of healthy controls or pwCF. (**B**) Purified CD25^+^CD127^–^ Tregs were stimulated with PMA/ionomycin and analyzed for the expression of Foxp3, Helios, CD137, CTLA4, LAP, and GARP by flow cytometry. (**C**) In vitro suppression assay with polyclonal Tregs from healthy controls or pwCF. Ex vivo–isolated Tregs were combined with proliferation dye–labeled allogeneic CD4^+^ Tresp cells at different Treg to Tresp ratios. Cells were stimulated polyclonally with CD3/CD28 beads. The percentage of inhibition of Tresp proliferation is shown for healthy controls (*n* = 5) and pwCF (*n* = 6). Right: Dot plot examples, with the numbers indicating the percentage of Tresp proliferation. (**D**) Percentage of memory cells within polyclonal CD25^+^CD127^–^ Tregs from healthy controls (*n* = 149) or pwCF (*n* = 200). (**E**) Representative dot plot examples for the parallel ex vivo detection of *A*. *fumigatus*–reactive CD4^+^ Tcons (CD154^+^) and Tregs (CD137^+^) by ARTE. PBMCs (1 × 10^7^) were stimulated with *A*. *fumigatus* or left unstimulated, and cell counts before and after combined magnetic CD154^+^CD137^+^ enrichment are indicated. (**F**) Overlayed flow-cytometric analysis of *A*. *fumigatus*–specific CD154^+^ and CD137^+^ cells. Numbers indicate the percentages among CD137^+^CD4^+^ T cells (light blue) and CD154^+^CD4^+^ T cells (dark blue). (**G**) *A*. *fumigatus*–reactive CD137^+^ and CD154^+^ Tmem cells (*n* = 3) were purified by FACS and analyzed for gene expression by real-time PCR. Data were normalized to GAPDH gene expression. (**H**) Frequencies of *A*. *fumigatus*–reactive CD137^+^ Tregs from healthy donors (*n* = 161) and pwCF (*n* = 135). (**I**) CD45RA and Ki-67 staining of *A*. *fumigatus*–reactive CD137^+^ Tregs. The percentage of marker-positive cells within CD137^+^CD25^+^ Tregs is indicated. (**J**) Percentage of memory cells within *A*. *fumigatus*–reactive CD137^+^ Tregs (healthy donors, *n* = 161; pwCF, *n* = 135). (**K**) Ki-67 expression of *A*. *fumigatus*–reactive CD137^+^ Tregs (healthy donors, *n* = 46; pwCF, *n* = 25). (**L** and **M**) TCR-β sequence analysis of the top 50 expanded *A*. *fumigatus*–specific Treg clones from healthy individuals (*n* = 4) and pwCF (*n* = 3). (**L**) Gini index depicting the distribution of TCR sequences. (**M**) Rényi diversity profiles. (**N**) In vitro suppression assay with ex vivo–isolated *A*. *fumigatus*–reactive Tregs. *A*. *fumigatus*–reactive CD137^+^ Tregs were combined with proliferation dye–labeled allogeneic responder CD4^+^ T cells (Tresp) at a Treg to Tresp ratio of 1:4. Cells were stimulated polyclonally with CD3/CD28 beads. The percentage of inhibition of Tresp proliferation is shown for healthy controls (*n* = 8) and pwCF (*n* = 7). Each symbol in **B**–**D**, **G**, **H**, **J**–**L**, and **N** represents 1 donor; horizontal lines indicate the mean in **B**–**D**, **H**, **J**, **K**, and **M**. Truncated violin plots with quartiles and range are shown in **G**. Box-and-whisker plots display the quartiles and range in **L**. Data indicate the mean ± SEM in **M**. Statistical differences were determined by 2-tailed Mann-Whitney *U* test in **B**, **D**, **H**, **J**, **K**, and **N** and by 2-tailed, unpaired *t* test in **L**.

We recently showed that Tregs dominate the CD4^+^ T cell response against environmental airborne antigens in healthy humans, including *A*. *fumigatus* spores (3, 4), and likely suppress the development of inflammatory Th2 responses. Lower numbers and reduced expression of suppressive cytokines in the total Treg population have previously been reported in pwCF (27, 29). Indeed, when we isolated total Tregs from PBMCs based on CD25^+^CD127^–^ expression (Figure 2A), we observed a trend toward reduced levels of Helios (*P* = 0.0774, 2-tailed Mann-Whitney *U* test) (Figure 2, A and B). In contrast, we did not observe differences in other Treg effector markers, such as CTLA4, LAP, or GARP (Figure 2B). However, polyclonal Tregs from pwCF had a less suppressive capacity when tested in an in vitro suppression assay, although the differences were not significant (Figure 2C and Supplemental Figure 1I). This might be explained by the observation that the total Treg compartment in pwCF contained fewer memory cells, but a higher fraction of naive-like cells, as determined by staining for CD45RA and CCR7 (Figure 2D).

We next analyzed whether *A*. *fumigatus*–reactive Tregs are also altered in pwCF, which could explain the development of the clonally expanded high-avidity Tmem response. Antigen-specific Tregs can be identified on the basis of expression of the activation marker CD137 (4-1BB) but a lack of CD154 expression after 7 hours of antigen activation (Figure 2E) (3, 30). Treg identity of the CD137^+^ cells following *A*. *fumigatus* stimulation was confirmed by coexpression of CD25 and FOXP3, but a lack of CD127 expression (Figure 2F). Multiplex real-time PCR analysis of FACS-purified *A*. *fumigatus*–reactive CD137^+^ cells showed high expression of the Treg-related genes *FOXP3*, *IKZF2* (Helios), *IL2RA* (CD25), and *ENTPD1* (CD39), but a lack of effector cytokines, such as *IL2*, *IFNG*, and *IL17A* (Figure 2G), confirming previous results (3). In contrast to CD154^+^ Tmem cells (Figure 1), we observed no significant differences in the frequencies of *A*. *fumigatus*–reactive Tregs between healthy individuals and pwCF (Figure 2H). The majority of *A*. *fumigatus*–reactive Tregs had a memory phenotype (Figure 2, I and J) and displayed basal expression of Ki-67 (Figure 2, I and K). This confirms their specific in vivo activation in response to continuous *A*. *fumigatus* exposure. We also observed no significant differences in the TCR repertoire of *A*. *fumigatus*–reactive Tregs between healthy donors and pwCF (Figure 2, L and M). Furthermore, in contrast to the total Treg compartment, *A*. *fumigatus*–reactive Tregs from pwCF showed no differences in their suppressive capacity in an in vitro suppression assay (Figure 2N and Supplemental Figure 1J).

In summary, these data indicate that *A*. *fumigatus*–reactive Tregs are not quantitatively or qualitatively altered in pwCF, whereas clonally expanded, high-avidity Tmem responses develop in CF.

### Different functional A. fumigatus–reactive T cell patterns develop in pwCF.

**Figure 3:**
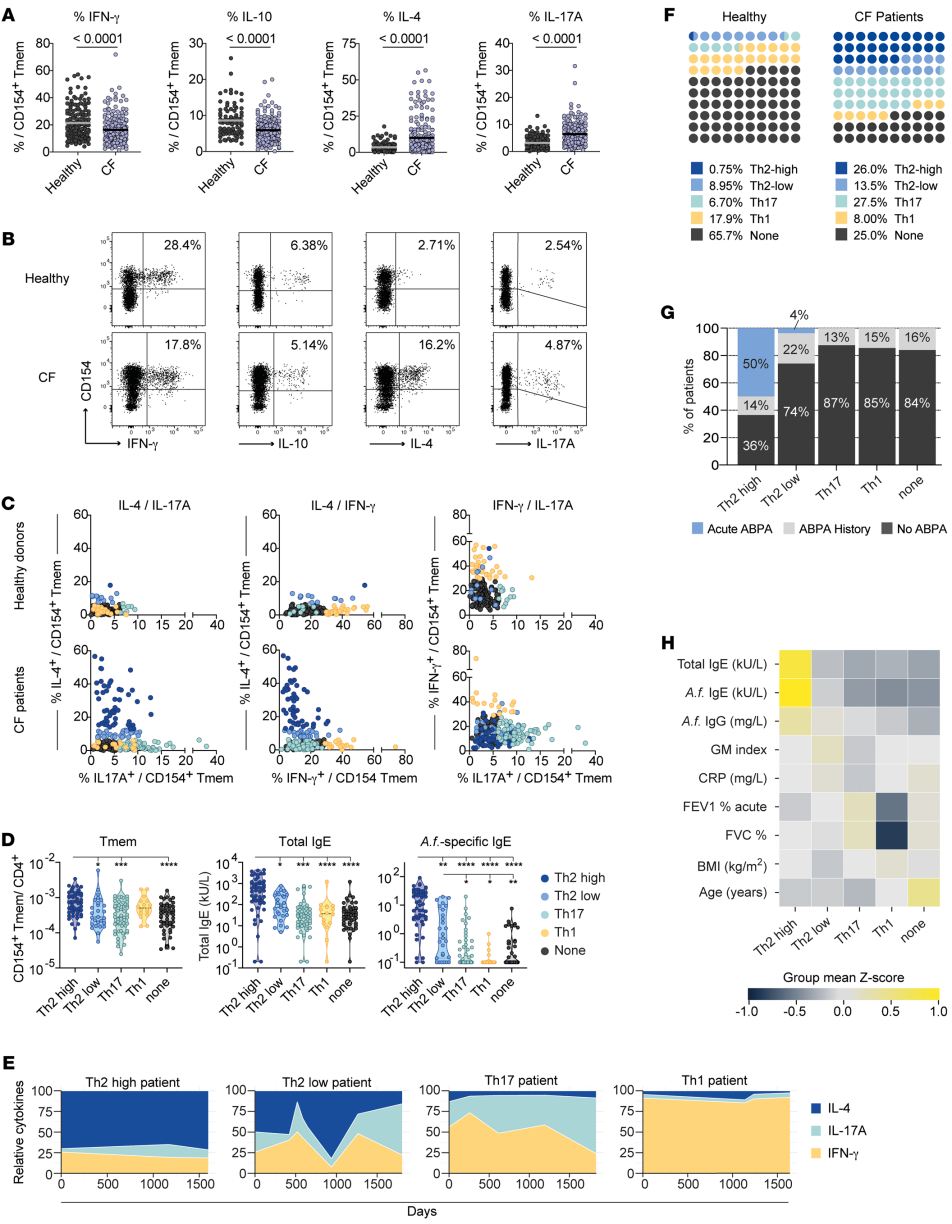
Different T cell reactivity pattern against *A*. *fumigatus* in pwCF. (**A**) Ex vivo cytokine production of *A*. *fumigatus*–reactive CD154^+^ Tmem cells from healthy donors (IFN-γ, *n* = 158; IL-17A, *n* = 158; IL-4, *n* = 134; IL-10, *n* = 87) and pwCF (*n* = 200). (**B**) Representative dot plots for ex vivo cytokine staining of *A*. *fumigatus*–stimulated cells following ARTE. The percentage of cytokine-producing cells within CD154^+^ Tmem cells is indicated. (**C**) Direct comparison of *A*. *fumigatus*–reactive cytokine production for healthy donors (*n* = 134) and pwCF (*n* = 200). Cut-off values for cytokine-producing cells within CD154^+^ Tmem cells were determined by ROC analysis (see Supplemental Figure 2). Donors whose cell percentages exceeded the cut-off are color coded (yellow: IFN-γ ≥29%; light green: IL-17A ≥6.1%; light blue: IL-4^lo^ ≥6.9%; dark blue: IL-4^hi^ ≥12.9%. (**D**) Frequencies of *A*. *fumigatus*–reactive Tmem cells, as well as total and specific IgE levels for pwCF within the different *A*. *fumigatus*–specific T cell reactivity groups. (**E**) Representative plots depicting the stability of the different *A*. *fumigatus*–specific cytokine reactivity patterns. *A*. *fumigatus*–reactive T cells of pwCF were monitored at least 4 times over a period of up to 4 years. Relative expression of IFN-γ, IL-17A, and IL-4 within the CD154^+^ Tmem population is shown. (**F**) Distribution of the different *A*. *fumigatus*–specific cytokine reactivity patterns for healthy donors and pwCF. The percentages of donors in each group are indicated. (**G**) Incidence of ABPA in the different *A*. *fumigatus*–specific T cell reactivity groups. The percentages indicate patients with acute ABPA, a history of ABPA, or who never had ABPA at the time the measurement was done. (**H**) Heatmap depicting the correlation of the different *A*. *fumigatus*–reactive T cell groups with clinical parameters. Values were *z* score normalized for each parameter and are plotted as the mean value for each T cell reactivity group. Each symbol in **A**, **C**, and **D** represents 1 donor; horizontal lines indicate the mean in **A**. Truncated violin plots with quartiles and range are shown in **D**. *P < 0.05, **P < 0.01, ***P < 0.001, and ****P < 0.0001, by 2-tailed Mann-Whitney *U* test (**A**) and Kruskal-Wallis test with Dunn’s post hoc test (**D**).

We next analyzed cytokine production by *A*. *fumigatus*–reactive Tmem cells to further characterize their functional capacities. In line with the frequent sensitization of pwCF to *A*. *fumigatus* antigens (3, 4, 21–25), *A*. *fumigatus*–reactive Tmem cells from pwCF showed increased production of IL-4, but also increased frequencies of IL-17A–producing cells. In contrast, IFN-γ and IL-10 production was significantly reduced (Figure 3, A and B).

We further asked whether these alterations in cytokine expression occur uniformly, or whether distinct reactivity patterns can be observed. To this end, we correlated the levels of cytokine producers for each individual patient (Figure 3C). Cutoff values for each cytokine were determined by receiver operating characteristic (ROC) analysis of healthy donors and patients, allowing discrimination of patients with 90% specificity (Supplemental Figure 2A). For IL-4 production, we further defined a second threshold to identify patients with particularly high IL-4 production (99% specificity) (Supplemental Figure 2A). Based on this, we identified subgroups of individuals whose anti–*A*. *fumigatus* response was dominated by elevated IL-4 levels (referred to herein as Th2-high or Th2-low responder), IFN-γ (Th1 responder), or IL-17A (Th17 responder) production, respectively (Figure 3C). Individuals who did not have elevated expression of IFN-γ, IL-17A, or IL-4 were categorized as the “non-cytokine” group. Th2-high patients showed the highest levels of total and *A*. *fumigatus*–specific IgE, as well as *A*. *fumigatus*–reactive Tmem cell frequencies (Figure 3D), whereas the Th2-low patients had an intermediate phenotype based on these parameters (Figure 3D). Despite the fact that Th1 responders and Th2-high patients had similarly high frequencies of *A. fumigatus*–reactive Tmem cells, the Th1 responders in general had low levels of total and specific IgE levels, as did the Th17 and non-cytokine responder groups. The different Th response patterns were also reflected by different expression levels of the chemokine receptors CCR4 and CCR6 and the Th2 marker CRTH2 on *A*. *fumigatus*–reactive Tmem cells (Supplemental Figure 2, B and C). These data indicate that a functionally diverse set of CD4^+^ Th cell phenotypes against *A*. *fumigatus* can develop in different pwCF. To analyze the stability of the observed reactivity pattern, we monitored more than 50 patients longitudinally over a period between 1 to 5 years (Figure 3E and Supplemental Figure 3). While Th2-high and Th1 patients showed a rather stable cytokine pattern over time, we observed more variability especially within the Th2-low group but also the Th17 patient group (Figure 3E), suggesting a less stably differentiated phenotype that might be actively modulated by acute confrontation with the fungus.

Comparison of the proportion of donors in each group revealed that the majority (>80%) of healthy individuals were in the non-cytokine (65.7%) or Th1 (17.9%) group (Figure 3F). These major reactivity patterns of healthy donors were strongly reduced in the CF cohort. Contrarily, pwCF displayed mainly Th2 (39.5%) and Th17 (27.5%) cytokine reactivity against *A*. *fumigatus*. In particular, the Th2-high phenotype was almost completely absent in healthy individuals (<1%), while 26% of pwCF presented this phenotype (Figure 3F). pwCF frequently develop ABPA, a severe *A*. *fumigatus*–driven complication with an unclear etiology. Difficulties in the diagnosis of ABPA occurs in CF because of overlapping clinical, radiological, microbiological, and immunological features often indistinguishable from other exacerbations (9, 10). Furthermore, it remains unclear why certain patients develop ABPA and others do not. Of note, patients with diagnosed acute ABPA were mainly in the Th2-high group, and, overall, 64% of patients in this group had either acute ABPA at the time of sampling or a history of ABPA (Figure 3G).

Further correlation of the different *A*. *fumigatus* reactivity groups with clinical parameters revealed lower lung function parameters in the Th1 responders (forced expiration volume [FEV1] of the Th1 patients was in mean of 41% versus 58% for the remaining cohort) (Figure 3H and Supplemental Table 1), as well as a higher number of Th1 patients with disease exacerbation at the time point of our measurement (Supplemental Figure 4). However, a higher number of disease exacerbations also applied to the Th2-low group. Interestingly, individuals in the non-cytokine group tended to be older than individuals in the other groups (non-cytokine mean age of 35.3 years vs. 27.6 years for the remaining cohort; non-cytokine vs. Th2-high *P* = 0.0190 vs. Th17 *P* = 0.0025 vs. Th2-low *P* = 0.0554 vs. Th1 *P* = 0.8256; Kruskal-Wallis test with Dunn’s post hoc test) (Figure 3H and Supplemental Table 1), suggesting that the lack of cytokine production might also be the result of T cell exhaustion due to long-term, chronic interaction with *A*. *fumigatus*.

In summary, these data show that the *A*. *fumigatus*–reactive CD4^+^ T cell response was also qualitatively altered in pwCF. These alterations were not uniform, but instead, functionally distinct anti–*A*. *fumigatus* reactivity patterns developed in individual patients. The Th2-high and Th1-reactivity patterns showed remarkable stability, indicating a long-term memory formation in response to *A*. *fumigatus*, while the other Th patterns might still have been actively modulated by acute exposure to the fungus.

### Th cell subsets selectively target different A. fumigatus proteins.

How the different effector reactivity patterns in pwCF arise remains unknown. Either T cells with the same specificity differentiate into multiple functional subtypes (31), or the specificity of the T cell and/or interaction with different fungal structures determines the differentiation pattern. We therefore analyzed whether the different *A*. *fumigatus*–reactive Th subsets have the same or different protein specificities.

### A.

**Figure 4:**
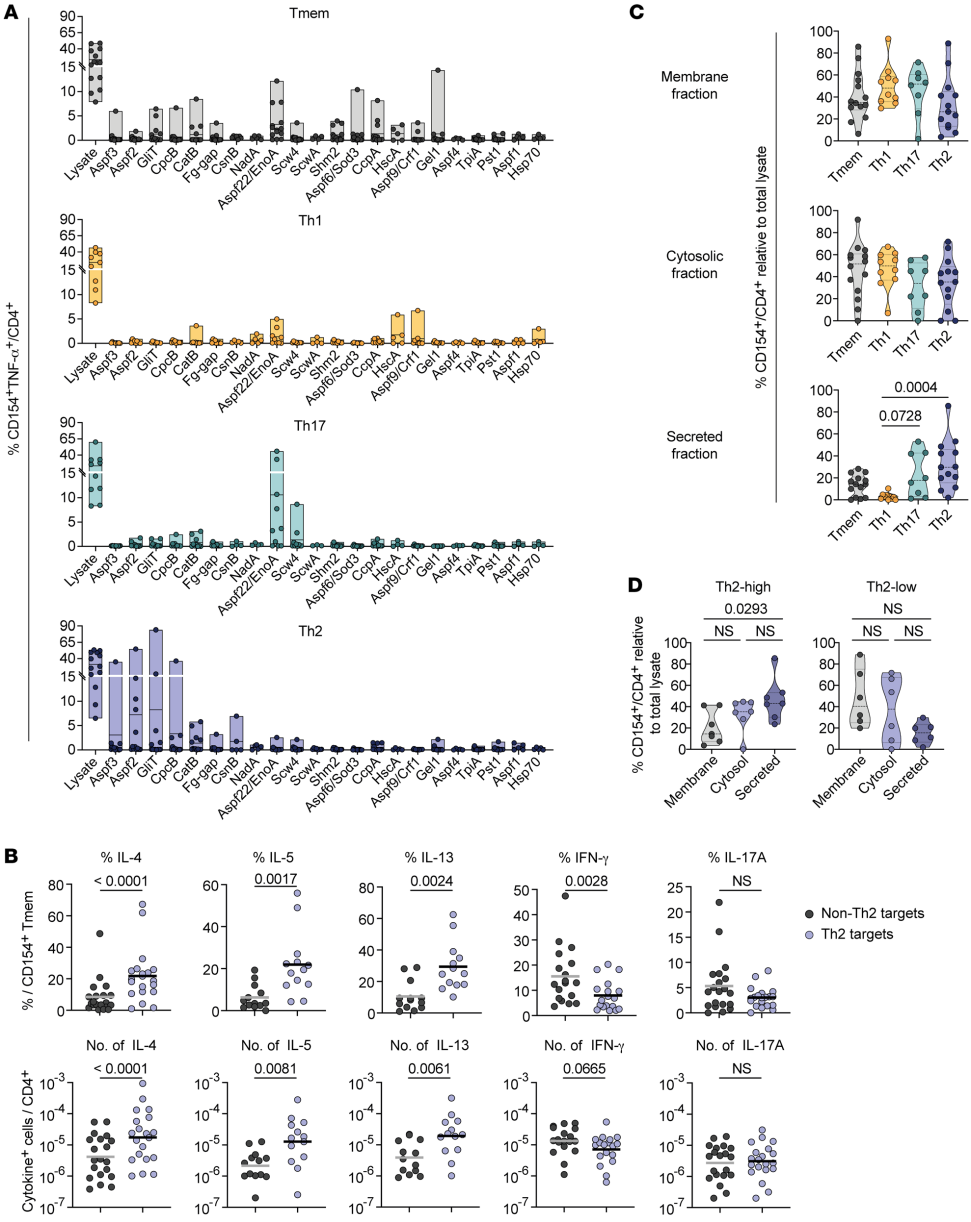
*A*. *fumigatus*–reactive Th cell subsets recognize different protein targets. (**A**) PBMCs from pwCF (*n* = 8–14) were stimulated with whole *A*. *fumigatus* lysate, and reactive CD154^+^ Tmem, Th1 (IFN-γ^+^), Th17 (IL-17A^+^), or Th2 (CRTH2^+^) cells were FACS sorted, expanded, and restimulated with a panel of single *A*. *fumigatus* proteins. Reactivity is indicated as the percentage of CD154^+^TNF-α^+^ within CD4^+^ T cells. (**B**) Th2 target proteins (Aspf2, Aspf3, CpcB, CatB, Fg-gap, GliT) and non-Th2 target proteins (Scw4, Aspf22, Pst1, Shm2, CcpA, TpiA, Crf1, Sod3) were pooled and used for ex vivo stimulation of PBMCs from *A*. *fumigatus*–sensitized pwCF. Relative cytokine production within reactive CD154^+^ Tmem cells (upper plots) and absolute frequencies of cytokine producers within CD4^+^ T cells (lower plots) are shown (IL-4, IFN-γ, IL-17A, *n* = 20; IL-5, IL-13, *n* = 13). (**C**) *A*. *fumigatus–*reactive T cell lines were generated as in **A** and restimulated with different *A*. *fumigatus* antigen extracts in the presence of autologous FastDCs derived from blood monocytes. Reactivity in relation to restimulation with the initially used total *A*. *fumigatus* lysate is shown. (**D**) Reactivity of expanded CD154^+^ Tmem cells from Th2-high (*n* = 7) or Th2-low (*n* = 6) patients to the different *A*. *fumigatus* protein fractions. Each symbol in **A**–**D** represents 1 donor; horizontal lines indicate the mean in upper plots and the geometric mean in lower plots of **B**; truncated violin plots with the quartiles and range are shown in **C** and **D**. Statistical differences were determined by 2-tailed, paired Wilcoxon rank test in **B**. and by Kruskal-Wallis test with Dunn’s post hoc test in **C** and **D**.

*fumigatus*–reactive total Tmem cells or IFN-γ^+^ Th1, IL-17A^+^ Th17, or CRTH2^+^ Th2 cells from pwCF were expanded and restimulated with a panel of more than 20 different *A*. *fumigatus* proteins (Figure 4A). In the total Tmem compartment, we observed low reactivity to multiple analyzed proteins, reflecting a mixed response to several protein targets. We did not identify dominant common protein targets of Th1 cells within our panel of single *A*. *fumigatus* proteins, but, rather, we detected weak reactivity against CatB, Aspf22, HscA, Crf1, and Hsp70 in individual patients (Figure 4A). In contrast, Th17 and Th2 cells displayed stronger reactivity to individual proteins that were mostly nonoverlapping. Th17 cells strongly reacted against Scw4 and Aspf22, whereas we identified the known allergens Aspf2 and Aspf3, but also GliT, CpcB, CatB, Fg-gap, and CsnB as new Th2 target proteins (Figure 4A). The generation of different Th cell responses by distinct *A*. *fumigatus* proteins was confirmed by an increased relative and absolute production of IL-4, IL-5, and IL-13 following ex vivo stimulation with pooled Th2 versus non-Th2 proteins (Figure 4B and Supplemental Figure 5). In contrast, IFN-γ and IL-17A production of *A*. *fumigatus*–reactive T cells in the same patients was mainly directed against the non-Th2 protein pool.

Since both the identified Th2 target proteins and the major Th17 target Aspf22 are highly abundant within the *A*. *fumigatus* secretome (32–34), we repeated the experiment using different subcellular fractions of the fungus for restimulation (Figure 4C). While total Tmem cells, and especially IFN-γ responses, were mainly directed against the membrane and cytosolic fraction, Th17 and Th2 cells also significantly reacted to secreted proteins, whereas reactivity to membrane and cytosolic fractions were rather reduced, especially in Th2 cells (Figure 4C), confirming our results with the single proteins. Interestingly, the separation of Th2 responders into Th2-high and Th2-low groups (see Figure 3) revealed a clear difference in the recognition of the secreted protein fraction occurring mainly by Th2 cells from the Th2-high group (Figure 4D). This suggests that the pathogenic Th2-high phenotype is driven by a restricted set of secreted *A*. *fumigatus* proteins.

In summary, these data indicate that distinct types of CD4^+^ T cell responses in pwCF develop against target proteins derived from different compartments of the fungus and suggest that secreted proteins may contribute to the pathologic alterations of Th17, and in particular Th2, responses in CF.

### A. fumigatus is a major inducer of anti-fungal Th2 responses in CF, but S. apiospermum–reactive Th2 cells expand in acute ABPA.

To further elucidate the potential drivers of the pathogenic Th2 response, we analyzed the contribution of other fungal pathogens. pwCF are typically colonized with multiple fungal microbes. Besides *A*. *fumigatus*, the most frequent isolated fungi from CF airways are the yeast *Candida albicans* and the mold *Scedosporium apiospermum* (*Pseudallescheria boydii*) (7, 8). Allergic sensitization and allergic bronchopulmonary mycosis (ABPM) caused by *C*. *albicans* or *S*. *apiospermum* have been discussed (35–37), but conclusive data are lacking.

**Figure 5:**
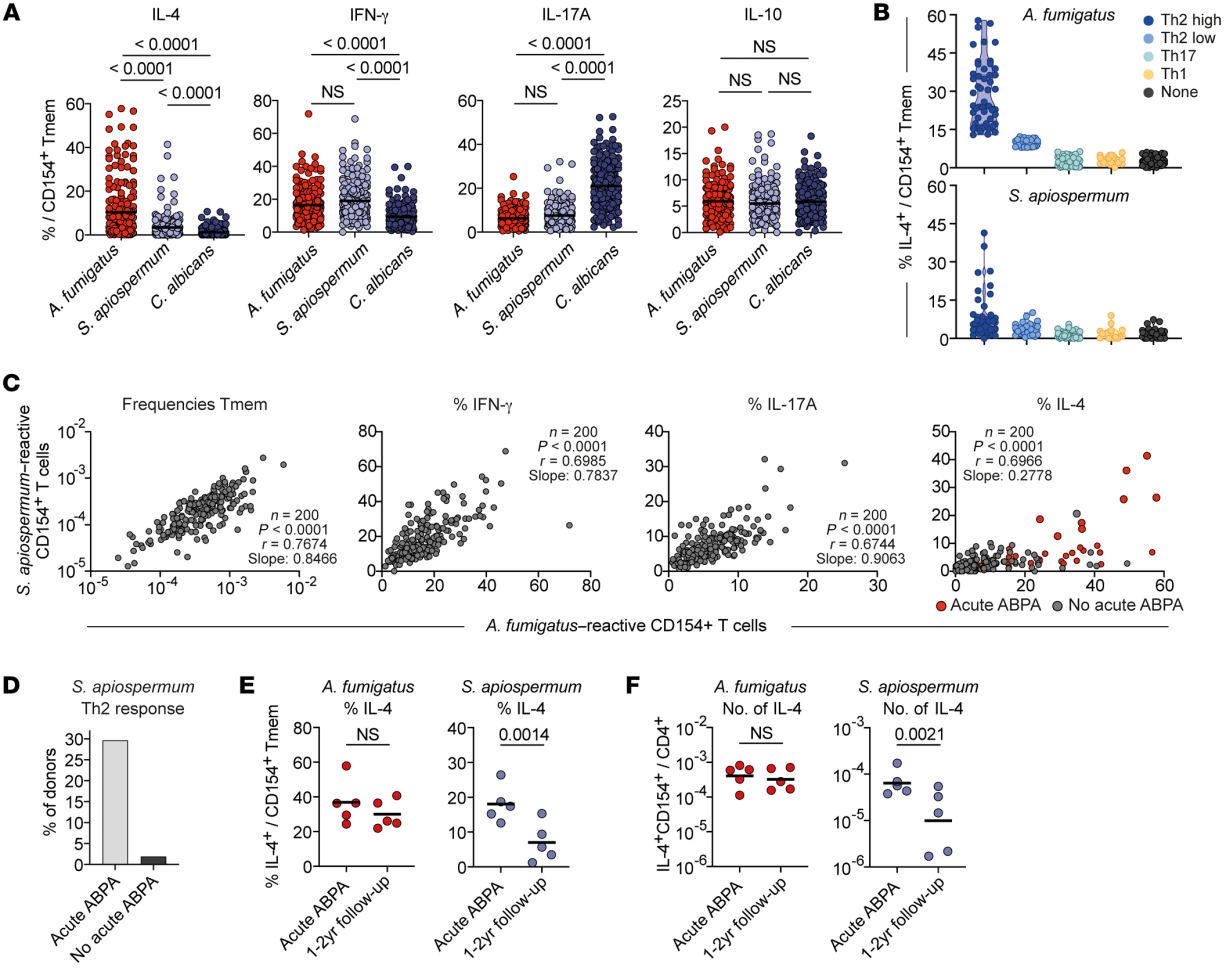
*A*. *fumigatus* is a major fungal Th2 driver in pwCF. (**A**) Ex vivo cytokine production of CD154^+^ Tmem cells with reactivity against different fungal pathogens in pwCF (*n* = 200). (**B**) IL-4 expression within *A*. *fumigatus*– or *S*. *apiospermum–*reactive Tmem cells of pwCF within the different *A*. *fumigatus*–defined T cell reactivity groups. (**C**) Spearman’s correlation between *A*. *fumigatus–* and *S*. *apiospermum*–reactive T cell frequencies and cytokine production. (**D**) Percentages of patients with or without acute ABPA among patients with an increased IL-4 response to *S*. *apiospermum*. (**E** and **F**) Five patients with acute ABPA and an increased Th2 response against *S*. *apiospermum* were reanalyzed after 1–2 years. (**E**) Relative IL-4 production within reactive CD154^+^ Tmem cells and (**F**) absolute frequencies of IL-4 producers within CD4^+^ T cells. Each symbol in **A**–**C**, **E**, and **F** represents 1 donor; horizontal lines indicate the mean in **A** and **E** and the geometric mean in **F**. Truncated violin plots with the quartiles and range are shown in **B**. Statistical differences were determined by Kruskal-Wallis test with Dunn’s post hoc test in **A** and by 2-tailed, paired *t* test in **E** and **F**.

**Figure 6:**
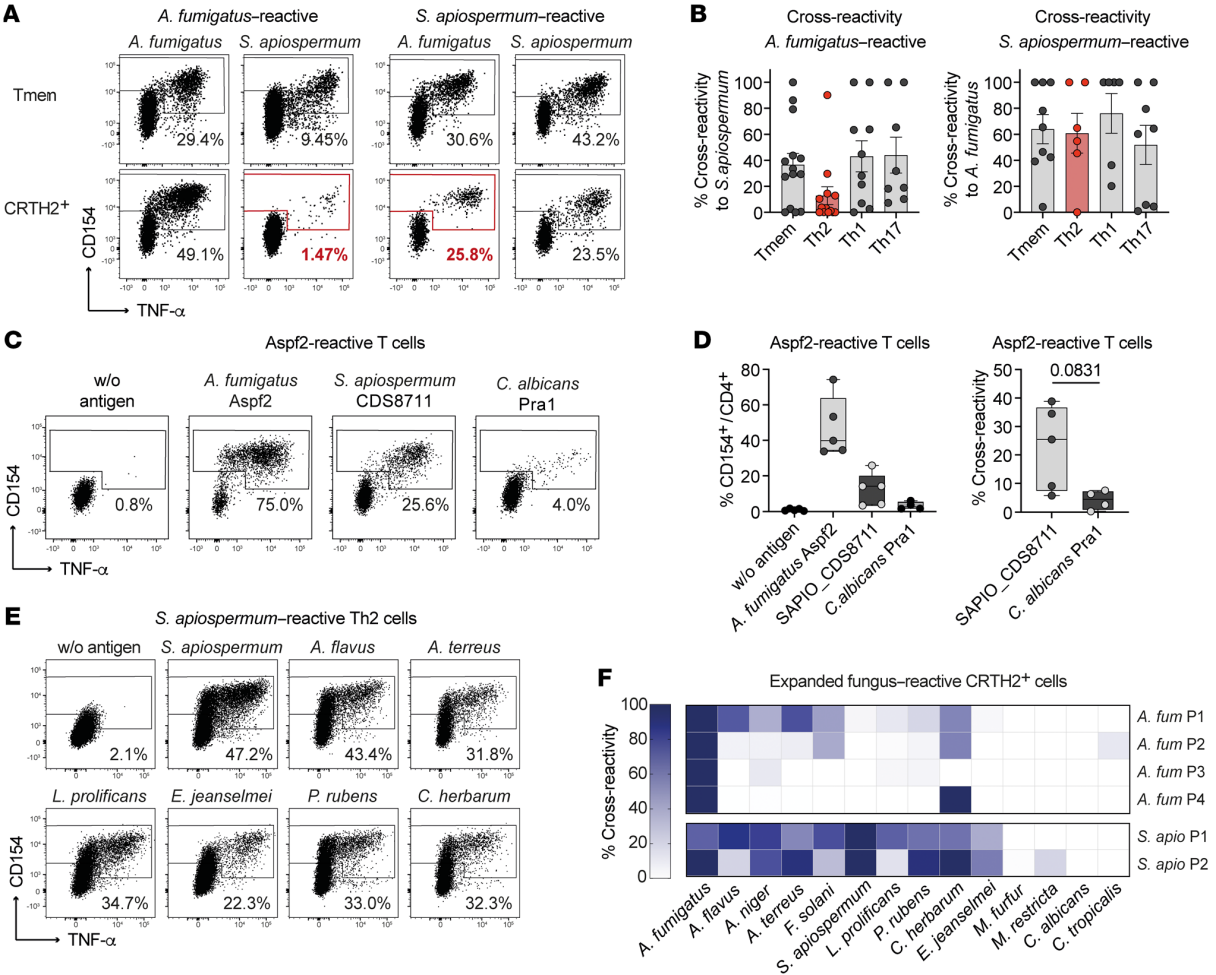
*A*. *fumigatus* is a major fungal Th2 driver in pwCF. (**A** and **B**) *A*. *fumigatus*–reactive (*n* = 8–14) or *S*. *apiospermum*–reactive (*n* = 6–9) CD154^+^ Tmem, Th1 (IFN-γ^+^), Th17 (IL-17A^+^), or Th2 (CRTH2^+^) cells from pwCF were FACS sorted, expanded, and restimulated with both fungal extracts. (**A**) Representative restimulation of expanded T cell lines. Percentages of CD154^+^TNF-α^+^ within CD4^+^ T cells are indicated in the plots. (**B**) Percentage of cross-reactivity of initially *A*. *fumigatus*–stimulated cells to *S*. *apiospermum* (left plot) and vice versa (right plot). Each symbol represents 1 donor, and data indicate the mean ± SEM. (**C** and **D**) PBMCs from Th2-high pwCF were ex vivo stimulated with *A*. *fumigatus* Aspf2. Reactive CD154^+^ Tmem cells were FACS purified, expanded, and restimulated in the presence of autologous FastDCs with Aspf2 or orthologous proteins from *S*. *apiospermum* (UniProtKD A0A084G096) or *C*. *albicans* (pH-regulated antigen Pra1 UniProtKB P87020). (**C**) Dot plot examples of restimulation. Cells were gated on CD4^+^ T cells, and the percentages of CD154^+^TNF-α^+^ T cells are indicated. (**D**) Percentage of cross-reactivity of Aspf2-reactive cells to the orthologous proteins of *S*. *apiospermum* and *C*. *albicans*. (**E** and **F**) Expanded *A*. *fumigatus*–reactive (*n* = 4) or *S*. *apiospermum–*reactive (*n* = 2) Th2 cells were restimulated with various fungal species. (**E**) Dot plot examples showing the restimulation of *S*. *apiospermum–*reactive Th2 cells with various fungal lysates. The percentages of CD154^+^ Th cells within CD4^+^ T cells are indicated in the plots. (**F**) The percentage of cross-reactivity in relation to total reactivity after restimulation with the specific fungal lysate is shown. Each row of the heatmap indicates 1 patient. Each symbol in **B** and **D** represents 1 donor; data indicate the mean ± SEM in **B**. Box-and-whisker plots display the quartiles and range in **D**.

Therefore, we also analyzed the T cell reaction against *C*. *albicans* and *S*. *apiospermum* in pwCF (Figure 5A). IFN-γ and IL-17A were readily produced also in response to these fungi. However, increased IL-4 production was mainly restricted to *A*. *fumigatus*–reactive cells but was occasionally found in *S*. *apiospermum*–specific T cells in individual patients (Figure 5A). Interestingly, all these patients also had high Th2 responses against *A*. *fumigatus* (Figure 5B), suggesting potential T cell cross-reactivity, which we have recently demonstrated to occur in humans between even distantly related fungal pathogens (18). To analyze a contribution of cross-reactivity between *A*. *fumigatus* and *S*. *apiospermum*, we correlated the frequencies, as well as cytokine production of reactive T cells against both fungi within the CF cohort. Indeed, the respective subsets of reactive Tmem cells, IFN-γ, and IL-17A were highly correlated for both fungal species (Figure 5C). In contrast, we observed a much lower regression slope for IL-4 production, with only 9 of 200 (4.5%) pwCF showing elevated IL-4 production against *S*. *apiospermum*, in addition to *A*. *fumigatus* (77 of 200 patients, 38.5%) (Figure 5C). Of note, 8 of these 9 patients had acute ABPA at the time point of our measurement, while 1 patient had a history of ABPA (Figure 6C, right). This resulted in almost 30% of patients with acute ABPA having increased Th2 responses to *S*. *apiospermum*, in addition to *A*. *fumigatus* (Figure 5D). For 5 of these patients, we had the opportunity to reanalyze them after 1–2 years. Although the relative and absolute values of *A*. *fumigatus*–reactive IL-4 remained stable, we observed a clear reduction of *S*. *apiospermum*–induced IL-4 production (Figure 5, E and F).

Overall, these data suggest that there was substantial T cell cross-reactivity between *A*. *fumigatus* and *S*. *apiospermum*. This cross-reactivity seemed to be strongly reduced within the Th2 compartment compared with the other *A*. *fumigatus*–reactive Th cell subsets. However, especially in patients with acute ABPA, *S*. *apiospermum–*reactive Th2 cells expanded, suggesting that deviation of Th2 responses to cross-recognized common fungal target proteins might be a risk factor for the development and/or exacerbation of ABPA.

### Cross-reactive Th2 cells can be triggered by various filamentous fungi.

To corroborate this hypothesis further, we isolated and expanded the *A*. *fumigatus–* and *S*. *apiospermum*–reactive T cell populations from pwCF (Supplemental Figure 6, A and B). Indeed, initially *A*. *fumigatus*–stimulated Tmem, IFN-γ, and IL-17A producers highly cross-reacted with *S*. *apiospermum* antigens and vice versa, probably due to a high sequence similarity of T cell target proteins (Figure 6, A and B). This cross-reactivity was clearly reduced within the *A*. *fumigatus*–reactive Th2 cells, pointing toward mainly *A*. *fumigatus*–restricted Th2 target proteins that are absent in *S*. *apiospermum*. However, by contrast, initially *S*. *apiospermum*–stimulated Th2 cells strongly cross-reacted with *A*. *fumigatus* (Figure 6, A and B). This asymmetric distribution of cross-reactive Th2 cells within the *A*. *fumigatus* versus *S*. *apiospermum* Th2 response suggests that in vivo Th2 induction is initiated by *A*. *fumigatus* but that cross-reactivity against other fungi may lead to selective expansion of preexisting cross-reactive Th2 clones under specific conditions, such as acute ABPA. To further confirm the cross-reactivity within the Th2 compartment on a protein level as well, we performed a sequence similarity search using BLAST and compared the *A*. *fumigatus* protein sequences with the *S*. *apiospermum* proteome. The identified Th2 target proteins of *A*. *fumigatus* showed between 45% and 91% identity to proteins from *S*. *apiospermum* (Table 1 and Supplemental Table 2). We selected the *A*. *fumigatus* protein Aspf2 as one of the strongest Th2 target proteins (Figure 4) and generated the closest orthologs from *S*. *apiospermum* (UniProtKB A0A084G096; 55% identity) and *C*. *albicans* (Pra1; UniProtKB P87020; 43% identity) as peptide pools spanning the whole protein sequence (Supplemental Figure 6C). Expanded Aspf2-reactive T cells from pwCF indeed showed cross-reactivity to the orthologous protein from *S*. *apiospermum*, but interestingly not to the *C*. *albicans* ortholog Pra1 (Figure 6, C and D), confirming our previous results with the whole fungal lysates.

To analyze whether cross-reactive Th2 cells recognize proteins also commonly present in other fungal species, we rechallenged expanded *A*. *fumigatus*– and *S*. *apiospermum–*reactive Th2 cells with a panel of different fungal species. As shown in Figure 6, E and F, *S*. *apiospermum–*reactive Th2 cells also showed remarkable cross-reactivity to distantly related filamentous fungi, including the classical allergy-inducing molds *Penicillium rubens* and *Cladosporium herbarum,* but not to yeasts. In contrast, within the *A*. *fumigatus* Th2 response, only low cross-reactivity was detected mainly against other *Aspergillus* species, but, interestingly, 3 of 4 patients also showed cross-reactivity to *C*. *herbarum*.

In summary, these data suggest that *A*. *fumigatus* is the major inducer of antifungal Th2 responses in pwCF. Th2 responses target a distinct set of *A*. *fumigatus*–specific proteins despite considerable T cell cross-reactivity between *A*. *fumigatus* and *S*. *apiospermum* within non-Th2 memory cell subsets. However, in a substantial number of patients with acute ABPA, we found that cross-reactive Th2 cells that recognized several, and even distantly related, fungal species expanded. Although these Th2 cells appeared to be initially primed by *A*. *fumigatus*, our data suggest that in vivo confrontation with various filamentous fungi, especially during acute ABPA, may have driven selective expansion of cross-reactive Th2 cells in pwCF in vivo.

## Discussion

Although fungal pathogens are frequently isolated from respiratory samples of pwCF, their clinical relevance for recurrent and chronic infections and inflammatory phenotypes of the lung remains poorly defined. By analyzing *A*. *fumigatus–*reactive Th cells in a large cohort of patients and healthy controls, we show that the majority of pwCF developed effector T cell responses indicating immune interaction with *A*. *fumigatus*. Still, we detected strong Treg responses against *A*. *fumigatus* in both healthy individuals and pwCF, confirming previous data (4). Within the effector Th cell compartment, we unraveled an unpredicted degree of heterogeneity, which is characterized by a variable contribution of Th1, Th2, and Th17 cells that can even coexist within individual patients. These different Th subsets react against different *A*. *fumigatus* protein fractions, suggesting that differential antigen uptake and presentation within distinct microenvironments direct Th cell subset development. The various Th reactivity patterns may contribute to the broad variety of clinical phenotypes and identify pathways for specific intervention. Indeed, patients with ABPA, the most severe *A*. *fumigatus*–related pathology in CF, display strong and stable Th2 responses with remarkable cross-reactivity against multiple filamentous fungi, suggesting that several fungal species may contribute to this severe complication.

We previously showed that in healthy individuals, the anti–*A*. *fumigatus* T cell response is dominated by Tregs, whereas conventional Th cells are largely naive (3, 4). This suggests that in healthy individuals, *A*. *fumigatus* is a major tolerogen rather than an immunogen, like other harmless aeroantigens, e.g., plant pollen or house dust mites (3, 38, 39). An impaired Treg compartment has previously been shown in pwCF (27, 29) and in particular in patients with ABPA (29). Consistent with these previous results, we found a lower suppressive capacity of polyclonal Tregs in pwCF. In contrast, the antigen-specific Treg response against *A*. *fumigatus* was not altered in pwCF. A possible explanation for the differences between polyclonal and *A*. *fumigatus*–reactive Tregs was an increased proportion of Tregs with a naive-like phenotype in pwCF, which merits further investigation.

We previously showed that in allergic pwCF, Th2 cells only develop against those proteins that are not targeted by specific Tregs (3). Thus in CF, new or higher quantities of antigens may become accessible to the immune system as a result of increased colonization of the CF lung with *A*. *fumigatus* and the formation of germinating conidia and hyphae (40), which secrete greater amounts of extracellular proteins and may lead to the priming of effector Th cell responses.

Our data identify an unpredicted high heterogeneity of Th1, Th2, and Th17 cells contributing to the anti–*A*. *fumigatus* immune response in pwCF. These different T cell subsets also coexisted within individual patients. How the different T cell reactivity pattern contributes to the broad spectrum of CF pathology and chronic inflammation and the control of fungal growth remains to be determined. Th2 and Th17 responses have especially been linked to lung inflammation in CF (41, 42). Interestingly, predominant Th1 and Th2-high cytokine patterns were remarkably stable over a period of up to 5 years. This indicates an *A*. *fumigatus*–specific imprinting of Th1 versus Th2 T cell fates. These data also suggest that patients who developed an *A*. *fumigatus*–specific Th1 cytokine profile were probably at low risk for developing ABPA. Indeed, the group of Th1 responders included 13% of patients with previous ABPA, whereas 64% of patients in the Th2-high group had either acute or prior ABPA. Thus, patients in the high-Th2 group but without a history of ABPA may represent a risk group for developing ABPA in the future and might benefit from close monitoring of symptoms. In addition, we also identified a subgroup of patients who exhibited intermediate Th2 cytokine and IgE phenotypes. These Th2-low patients, as well as patients with a predominant Th17 phenotype, displayed more variability in the cytokine response over time. Such an unstable T cell response may still be shifted in one direction or the other, which may offer the possibility of therapeutic intervention.

Another open question is how different Th reactivity patterns can develop against a single pathogen (43) and even within the same individual, as we describe here. One possible mechanism is antigen compartmentalization, meaning that antigens are physically separated into different microenvironments fostering different Th subset development. Previous data have shown that *A*. *fumigatus*–specific Tregs can be detected in virtually all healthy donors and are strongly directed against fungal membrane proteins (19). Thus, *A*. *fumigatus* membrane antigens seem to be commonly accessible to the immune system for antigen presentation to drive T cell reactions. In pwCF, Th1 cells also recognized membrane and cytosolic proteins, suggesting similar antigen uptake and presentation for both subsets. Recognition of the same target fractions by Th1 cells and Tregs might also explain why patients with Th1-dominated responses represented the smallest group in the CF cohort (<10%), as effector priming against such antigen targets may have been suppressed by Tregs.

In contrast, the most highly increased effector cell subsets in pwCF, i.e., Th2 and Th17 cells, were also strongly directed against secreted fungal proteins. Increased fungal protein secretion is linked to *A*. *fumigatus* pathogenesis (44), and thus secreted proteins may occur in higher quantity in the CF lung compared with the lungs of healthy individuals. Secreted proteins, e.g., proteases (45), might also be able to cross the epithelial barrier without fungal invasion and thus may be taken up by different antigen-presenting cells as compared with membrane and cytosolic proteins that remain bound to fungal bodies. Recognition of secreted proteins by Th2 cells was also confirmed for the individual proteins identified in the study, which include known allergens, such as Aspf2 and Aspf3 (46, 47), but also the new Th2 target proteins GliT, CpcB, CatB, Fg-gap, and CsnB, all of which are highly abundant in the secretome of *A*. *fumigatus* (32–34). Another factor that determines the heterogeneity of Th cell functionality is T cell cross-reactivity. We recently showed that *A*. *fumigatus* itself does not generate Th17 responses in humans, but rather recruits *C*. *albicans–*specific Th17 cells via cross-reactivity to homologous peptides (18). In line with that, the Th17 target proteins Scw4 and Aspf22 identified here share high sequence similarity with *C*. *albicans* (18). This cross-reactivity to *C*. *albicans* might also contribute to the longitudinal variability of the Th17 responder group, which may be influenced by variable exposure to *C*. *albicans*, as we showed before (18).

Here, we unraveled another T cell cross-reactivity between *A*. *fumigatus* and the distantly related filamentous fungi *S*. *apiospermum*, which may amplify ABPA. *S*. *apiospermum* is the second most common filamentous fungus in respiratory samples from pwCF (7, 48, 49). We identified cross-reactive Th2 cells that reacted not only to *S*. *apiospermum* but also other filamentous fungi, but not to more distantly related yeasts, in one-third of patients with acute ABPA. In contrast, in individuals with nonacute ABPA, Th2 cells reacted only to *A*. *fumigatus* without cross-reactivity to *S*. *apiospermum*. Indeed, Th2 cells were absent in *S*. *apiospermum–* or *C*. *albicans–*reactive T cells in the majority of pwCF. This suggests that *A*. *fumigatus* has a dominant Th2 priming capacity involving specialized host-fungus interactions and/or antigen delivery pathways and most likely a high antigen dose due to colonization of the lung. Once established, *A*. *fumigatus*–specific Th2 cells can then be expanded via exposure to other fungal species, leading to the outgrowth of interspecies cross-reactive Th2 cells. Interaction with these other fungi may thus actively contribute to the manifestation and/or exacerbation of acute ABPA. The selective reduction of *S*. *apiospermum*–specific, but not *A*. *fumigatus*–specific, Th2 cells upon resolution of acute ABPA supports this hypothesis. Triggering of cross-reactive Th2 responses by other common filamentous fungi may also explain the discrepancy between the detection of *A*. *fumigatus* in only 40%–60% of sputum cultures of patients with diagnosed acute ABPA (40). We confirmed T cell cross-reactivity on the protein level using Aspf2, one of the strongest Th2 target proteins of *A*. *fumigatus*, and its closest ortholog from *S*. *apiospermum*, indicating that *S*. *apiospermum* proteins can activate *A*. *fumigatus–*primed Th2 cells. Further identification of cross-recognized proteins of exaggerated Th2 responses in ABPA, as well as in non-ABPA fungal allergy, may be a promising strategy to develop improved diagnostic assays (50) and to enable specific targeting of cross-reactive Th2 cells as a therapeutic option.

In summary, we provide a unique example of the role of antigen compartmentalization and antigen specificity in driving distinct T cell reactivity patterns against a single fungal pathogen. The dissection of heterogenous Th cell reactions and their target antigens in pwCF can facilitate the development of targeted therapeutic strategies.

## Methods

### Fungal lysates and proteins.

Fungal lysates were generated by methods appropriate for each organism, as previously described (18). In brief, conidia from *Aspergillus fumigatus* (ATCC 46645) and *Scedosporium apiospermum* (Fungiscope 298) were grown for 7 days on malt agar plates and germinated in *Aspergillus* minimal medium (AMM). Mycelia were collected by Miracloth, washed with ultrapure water, disrupted in liquid nitrogen by grinding using a mortar and pestle, and diluted with PBS. Lyophilized extracts of *Candida albicans* (SN-1 6946) were purchased from Greer Laboratories and reconstituted with sterile water at a protein concentration of 1 mg/mL. Lysates were stored at –20°C in aliquots until use. All fungal lysates were used in a concentration of 40 μg/mL for stimulation.

Peptide pools of the *A*. *fumigatus* proteins SHMT (Shm2), Aspf3 (Pmp20), CatB, Gel1, Sod3 (Aspf6), Crf1 (Aspf9), and Aspf22 (EnoA) were purchased from Miltenyi Biotec and used in a concentration of 0.6 nmol/peptide/mL. Peptide pools of the *S*. *apiospermum* protein UniProtKB A0A084G096 and the *C*. *albicans* protein pH-regulated antigen Pra1 UniProtKB P87020 were purchased from peptide&elephants GmbH. Recombinant Aspf1 was purchased from Indoor Biotechnologies. Production of recombinant Aspf2, CpcB, Fg-gap, TpiA, GliT, Pst1, and Scw4 is described in ref. 19, CcpA in ref. 51, CsnB in ref. 52, Aspf4 in ref. 53, and HscA and Hsp70 in ref. 54. Recombinant NadA (55) was provided by Matthias Ziegler (University of Bergen, Bergen, Norway). For *A*. *fumigatus* ScwA, a synthetic gene (Invitrogen GeneArt) coding for ScwA (amino acids 19–93) was expressed as N-terminal maltose-binding protein (MBP) fusion separated by a tobacco etch virus (TEV) protease site in *E. coli* BL21 (DE3), via a modified pET28a vector (Novagen). The MBP-ScwA fusion protein was produced in *E*. *coli* BL21 (DE3) cells by autoinduction (Overnight Express Instant TB Medium, Novagen) at 30°C. Harvested cells were lysed in buffer A (20 mM Tris/HCl, 200 mM NaCl, pH 7.5) at 1,000 bar using a high-pressure homogenizer (Emulsiflex C5, Avestin). Cleared bacterial lysate was loaded onto a 50 mL Dextrin Sepharose High Performance column (Cytiva). The protein was eluted with buffer A containing 10 mM maltose, and the fusion protein was digested with TEV protease overnight at 22°C. For removal of the C-terminal His-tagged MBP and His-tagged TEV protease, the digested protein was loaded onto a 20 mL Ni Sepharose 6 Fast Flow column (Cytiva). The flow-through fraction containing tag-less ScwA was further purified by size exclusion chromatography (SEC) on a Superdex 75 prep grade 26/600 column (Cytiva) in 50 mM NaH2PO4 and 150 mM NaCl (pH 7.5).

Proteins were stored in 1× PBS at –20°C and used in a final concentration of 10 μg/mL for stimulation.

### Antigen-reactive T cell enrichment.

PBMCs were freshly isolated from EDTA blood samples on the day of blood donation by density gradient centrifugation (Biocoll; Biochrom). ARTE was performed as previously described (3, 18–20, 56). In brief, depending on the available number of cells, 0.5 × 10^7^ to 2 × 10^7^ PBMCs were plated in RPMI-1640 medium (Gibco, Thermo Fisher Scientific) supplemented with 5% (v/v) human AB serum (MilliporeSigma) at a cell density of 1 × 10^7^ PBMCs/2 cm^2^ in cell culture plates and stimulated for 7 hours in the presence of 1 μg/mL CD40 and 1 μg/mL CD28 pure antibody (both from Miltenyi Biotec). Brefeldin A (1 μg/mL) (MilliporeSigma) was added for the last 2 hours. Cells were labeled with CD154-biotin followed by anti-biotin MicroBeads (CD154 MicroBead Kit; Miltenyi Biotec) and magnetically enriched by 2 sequential MS columns (Miltenyi Biotec). Surface staining was performed on the first column, followed by fixation and intracellular staining on the second column. For parallel detection of *A*. *fumigatus*–specific Tregs, cells were labeled with CD154-biotin and CD137-phycoerythrin (CD137-PE), followed by anti-biotin and anti-PE MicroBeads (CD154 and CD137 MicroBead Kit; both from Miltenyi Biotec), and then magnetically isolated as described above. In some experiments, antigen presentation was blocked by addition of an HLA-DR antibody (50 μg/mL; clone AC122; Miltenyi Biotec) during the 7-hour stimulation.

Frequencies of antigen-specific T cells were determined on the basis of the total count of CD154^+^ Tcons and CD137^+^ Tregs after enrichment, normalized to the total number of CD4^+^ T cells applied on the column. For each stimulation, background cells enriched from the nonstimulated control were subtracted.

### Flow cytometry.

Cells were stained using different combinations of fluorochrome-conjugated antibodies: CD4-APC-Vio770 (M-T466), CD8-VioGreen (REA734), CD14-VioGreen (REA599), CD20-VioGreen (LT20), CD154-FITC (REA238), Ki-67-Vio667 (REA183), IL-17A–PE–Vio770 (CZ8-23G1), CRTH2-PE-Vio770 (REA598), CCR4-APC (REA279), IL-5–VioR667 (JES1-39D10), IL-13–PE (JES10-5A2.2), CD137-PE (4B4-1), CD4-FITC (REA623), CD154-APC (5C8), TNF-α–PE–Vio770 (cA2), CD127-FITC (REA614), CD25-APC (4E3), LAP-PE (REA1214), GARP–VioBright B515 (REA166) (all from Miltenyi Biotec); CD45RA-PE-Cy5 (HI100), IFN-γ–BV785 (4S.B3), IL-4–BV605 (MP4-25D2), IL-10–PE–Dazzle (JES3-9D7), CCR6-BV421 (G034E3), IL-4–PerCP–Cy5.5 (MP4-25D2), CD25-BV421 (BC96), CD127-BV605 (A019D5), Foxp3-AF647 (206D), Helios PE–Cy7 (22F6), and CTLA4-BV605 (BNI3) (all from BioLegend); and IL-2–BV711 (5344.111; BD Biosciences). Viobility 405/520 Fixable Dye (Miltenyi Biotec) was used to exclude dead cells. Surface staining of chemokine receptors was performed for 30 minutes at room temperature. For intracellular staining cells were fixed and permeabilized with the Inside Stain Kit (Miltenyi Biotec). Staining for FOXP3 was performed using the Foxp3 Staining buffer Set (Miltenyi Biotec).

Data were acquired on a LSR Fortessa (BD Biosciences). High-throughput screening of expanded T cell lines in 384-well plates was performed on a MACSQuantX Analyzer (Miltenyi Biotec). FlowJo software (Treestar) was used for analysis.

### In vitro expansion and restimulation of antigen-reactive T cell lines.

*A*. *fumigatus*–reactive CD154^+^ T cells were FACS purified and expanded in the presence of 1:100 autologous antigen-loaded irradiated feeder cells in expansion medium (TexMACS medium; Miltenyi Biotec), supplemented with 10 % (v/v) human AB-serum (GemCell), 200 U/mL IL-2 (Proleukin; Novartis), 100 IU/mL penicillin, 100 μg/mL streptomycin, 0.25 μg/mL amphotericin B (Antibiotic Antimycotic Solution; MilliporeSigma), 20 μM β-mercaptoethanol (Gibco, Life Technologies, Thermo Fisher Scientific), 2 mM glutamine, and 30 ng/mL anti-CD3 (OKT-3; Miltenyi Biotec) at a density of 2.5 × 10^6^ cells/cm^2^. During expansion for 2–3 weeks, medium was replenished and cells were split as needed.

For expansion of cell lines from different T cell subsets, PBMCs were stimulated with *A*. *fumigatus* or *S*. *apiospermum* for 5 hours. Cells were labeled with IFN-γ and IL-17A catch reagent (Cytokine secretion assay; Miltenyi Biotec) and incubated for an additional 45 minutes in RMPI-1640 medium supplemented with 2% (v/v) human AB serum under continuous rotation. Cells were further labeled with CD154-biotin, followed by anti-biotin MicroBeads and magnetically enriched using MS columns (all from Miltenyi Biotec). Cells were stained on the column with IL-17A–Vio667, IFN-γ–FITC, CRTH2-PE-Vio770, CD45RA-Viogreen, and anti–biotin-PE (all from Miltenyi Biotec). Antigen-specific Tmem cells or CRTH2^+^, IFN- γ^+^, or IL-17A^+^ T cells were FACS sorted in bulk into 96-well, round-bottomed plates with 1 × 10^5^/well irradiated autologous feeder cells in expansion medium.

For restimulation, FastDCs were generated from autologous CD14^+^ MACS-isolated monocytes (CD14 MicroBeads; Miltenyi Biotec) by cultivation in X-Vivo 15 medium (BioWhittaker/Lonza), supplemented with 1,000 IU/mL GM-CSF and 400 IU/mL IL-4 (both from Miltenyi Biotec). Before restimulation, expanded T cells were rested in RPMI-1640 plus 5% human AB serum for 2 days. Expanded T cells (5 × 10^4^) were restimulated at a ratio of 1:1 with antigen-loaded FastDCs in 384-well, flat-bottomed plates for 7 hours, with addition of 1 μg/mL brefeldin A (MilliporeSigma) for the last 4 hours.

### Analysis of functional avidity.

In vitro–expanded *A*. *fumigatus*–specific T cells were rechallenged with decreasing antigen concentrations (100, 50, 10, 1, 0.1, and 0.01 μg/mL) in the presence of autologous FastDCs generated from blood monocytes and analyzed for reexpression of CD154 and cytokines. Antigen concentrations required for a half-maximal response (EC_50_ values) were calculated from dose-response curves using GraphPad Prism (GraphPad Software). These curves were plotted as a semilogarithmic plot, in which the amount of antigen was plotted (on the *x* axis) as the log of antigen concentration, and the response was plotted (on the *y* axis) using a linear scale. To compare the EC_50_ values of cells from different donors, the bottom and top of the curve were defined as 0% and 100%, respectively.

### Polyclonal Treg analyses.

Polyclonal Tregs were isolated from PBMCs using the CD25^+^CD127^dim^ isolation Kit (Miltenyi Biotec). The purity of isolated cells was analyzed by staining for CD25, CD127, Foxp3, and Helios using the Foxp3 Staining Buffer Set (Miltenyi Biotec). Tregs (5 × 10^4^) were stimulated in 384-well, flat-bottomed plates for 24 hours with PMA/ionomycin (MilliporeSigma). Stimulated cells were stained for CD137, CTLA4, LAP, and GARP and analyzed by FACS.

### Treg suppression assays.

CD4^+^ responder T (Tresp) cells were isolated with the CD4^+^ untouched T cell Isolation Kit (Miltenyi Biotec) and labeled with the CellTrace Violet Cell Proliferation Kit (Invitrogen, Molecular Probes) at a final concentration of 2.5 mM.

Tresp cells (5 × 10^4^) were cocultured at different ratios with ex vivo–isolated polyclonal or *A*. *fumigatus*–specific CD137^+^ Tregs or CD154^+^ cells as a control in 384-well plates in RPMI-1640 supplemented with 5% AB serum. Cells were stimulated polyclonally with anti-CD3/-CD28 beads (Treg Suppression Inspector; Miltenyi Biotec). On day 6, dilution of proliferation dye and expression of CD25 (Miltenyi Biotec) were analyzed by flow cytometry. Allogenic Tresp cells were discriminated from Tregs via discordant HLA-A2 expression.

### Gene expression and data analysis.

A total of 400 *A*. *fumigatus*–reactive Tregs and Tmem cells were sorted in duplicates directly into 96-well twin.tec PCR plates (Eppendorf) containing 5 μL 2× Reaction Mix (SuperScript III One-Step RT-PCR System) and 0.1 μL SUPERase-In RNase inhibitor (both from Invitrogen, Thermo Fisher Scientific). Gene expression was analyzed simultaneously with the 96.96 Dynamic Array Integrated Fluidic Circuits from Fluidigm. Preamplification of genes by reverse transcription and cDNA synthesis (18 cycles) was performed with the Cells Direct One-Step qPCR kit (Life Technologies, Thermo Fisher Scientific) and TaqMan gene expression assay mix (Applied Biosystems). Data were normalized to GAPDH, and mean values of the duplicates were used.

### Adaptive immune receptor repertoire–based (AIRR-Seq–based) TCR analysis.

Genomic DNA was isolated from FACS-purified *A*. *fumigatus*–specific CD154^+^ memory T cells using the AllPrep DNA/RNA Micro Kit (QIAGEN). The recombined TCR-β locus was amplified as previously described (57). Library preparation and sequencing were performed using Illumina MiSeq Technology at the NGS core facility of the Berlin Brandenburger Center for Regenerative Therapies (Berlin, Germany). Reads with an average quality score below 30 were excluded from the analysis. The remaining high-quality reads were processed using IMSEQ (58). Each clonotype was assigned an ID including Vβ- and Jβ-segment identity as well as CDR3 amino acid sequence.

The equality of the distribution of TCR-β sequences was calculated for the top 50 expanded clones using the Gini index (59). The index ranges from 0 to 1, where 0 is total equality, i.e., all clones have the same proportion, and 1 is total inequality, i.e., a population dominated by a single clone. Clonal diversities of the TCR-β repertoires were evaluated for the top 50 expanded clones using Rényi diversity profiles (60). By varying the α parameter, different diversity indices are calculated at α = 0, 1, 2, and infinite; the profiles provide the logarithm of richness, the Shannon diversity, the reciprocal Simpson diversity, and the Berger-Parker index, respectively. This means that the sample with the highest value at α = 0 has the highest richness but that the lower value at α = infinite indicates higher proportion of the most abundant sequence. A sample with a profile that is overall higher than the profiles of other samples is more diverse.

### Software.

Flow cytometric data were analyzed using FlowJo (Treestar) software. Graphics and statistics were created with GraphPad Prism 8.4.3 (GraphPad Software). The TCR diversity analysis was performed using R, version 3.3.2. Rényi diversity profiles were calculated using the R package ‘vegan,’ version 2.3-2 (61). The Gini indices were calculated using the R package ‘ineq,’ version 0.2-13 (62).

### Statistics.

Statistical parameters including the exact value of *n*, the definition of center, dispersion, and precision measure, and statistical significance are reported in the Figures and the legends. Two-tailed Mann-Whitney test, 2-tailed unpaired *t* test, Kruskal-Wallis test with Dunn´s post hoc test, or 2-tailed paired Wilcoxon rank test were used to determine significances. Statistical tests were performed with GraphPad Prism 8.4. Statistical tests were selected on the basis of appropriate assumptions with respect to data distribution and variance characteristics. *P* values of less than 0.05 were considered statistically significant.

### Study approval.

Peripheral blood samples from healthy controls were obtained from blood bank donors of the DRK (Dresden, Germany), the Charité blood bank (Charité Universitätsmedizin Berlin, Germany), the Institute for Transfusion Medicine, UKSH (Kiel, Germany), and from in-house volunteers (ethics committee Charité Berlin EA1/272/15; ethics committee CAU Kiel D578/18). Peripheral EDTA blood samples from pwCF were obtained from the Department of Pediatric Pneumology and Immunology of the Cystic Fibrosis Center Berlin (Charité Universitätsmedizin Berlin, Germany), and from the Cystic Fibrosis Center Potsdam (Westbrandenburg Clinic Potsdam, Germany) (ethics committee Charité Berlin EA1/290/13; EA1/149/12; EA1/272/15, ethics committee Potsdam AS48(bB)/2021). Inclusion criteria for pwCF were as follows: confirmed diagnosis of CF; age between 6 and 70 years; ability to perform a pulmonary function test; ability to produce sputum samples for microbiological evaluation and detection; ability to undergo blood sample collection; provision of written informed consent. Acute ABPA was defined according to the consensus guidelines (63) and a history of ABPA was defined as no recurrence of ABPA for more than 6 months after treatment cessation. All blood donors gave written informed consent prior to participation in the study.

## Author contributions

PB and AS conceptualized the study. PB, HS, TH, CI, LL, US, LL, and NB performed experiments. PB, CS, PE, ET, and ER conducted formal analyses. HW, FE, LJJ, MGB, PH, SB, BH, AAB, and OK provided resources. Patient recruitment and clinical data: CS, PE, CG, JR, ES, MS, and JGM were responsible for patient recruitment and clinical data collection. PB and AS acquired funding. All authors provided discussion, participated in revising the manuscript, and agreed to the final version.

## Supplementary Material

Supplemental data

ICMJE disclosure forms

## Figures and Tables

**Table 1:**
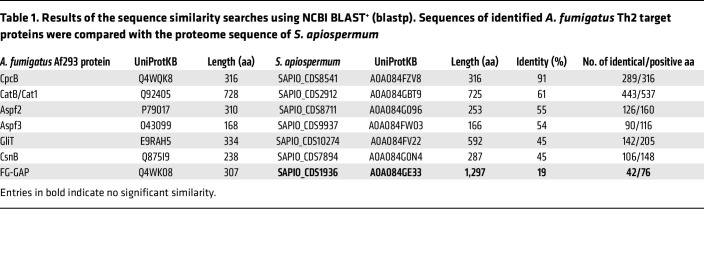
Results of the sequence similarity searches using NCBI BLAST^+^ (blastp). Sequences of identified *A. fumigatus* Th2 target proteins were compared with the proteome sequence of *S. apiospermum*

## Version 1 01/26/2023

In-Press Preview

## Version 2 03/01/2023

Electronic publication
