# Supplementary material for: Antigen specificity and cross-reactivity drive functionally diverse anti–Aspergillus fumigatus T cell responses in cystic fibrosis
Source: J Clin Invest. 2023 Mar 1;133(5):e161593. doi: 10.1172/JCI161593 (PMC9974102; doi:10.1172/JCI161593)
Supplement: Supplemental data [file jci-133-161593-s138.pdf]

Supplemental Figure 1

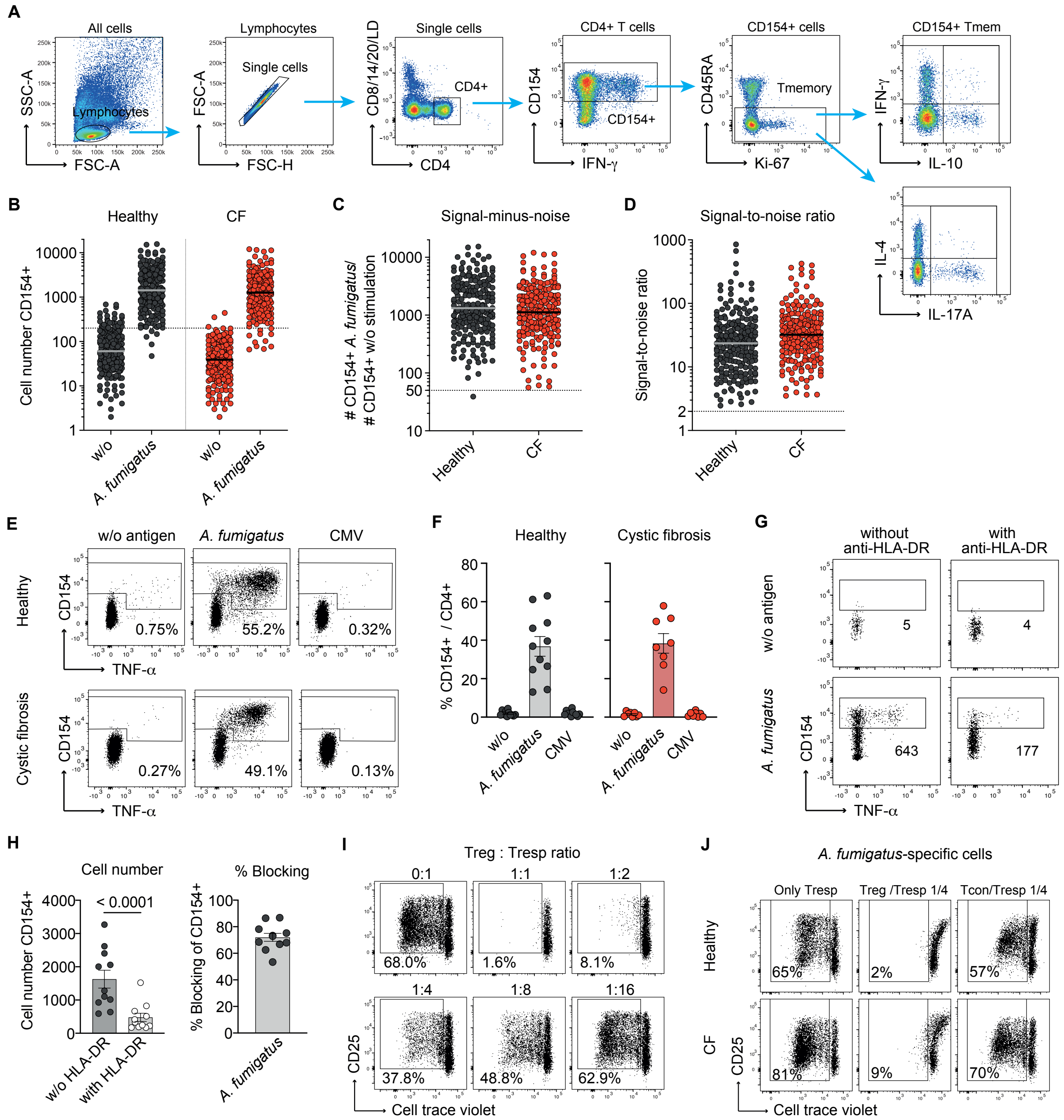

Figure S1. Specificity of CD154 induction on CD4+ T cells following stimulation with *A. fumigatus* lysate.

(A) Gating strategy following enrichment of CD154+ cells via ARTE. **(B)** Absolute cell counts of CD154+CD4+ T cells obtained by ARTE from unstimulated control or *A. fumigatus* lysate stimulated samples, respectively. **(C)** Signal-minus-noise numbers of enriched CD154+ cells. For each donor, background cells enriched from the non-stimulated control were subtracted and the absolute numbers of obtained *A. fumigatus*-reactive CD154+ T cells are shown. **(D)** Signal-to-noise ratio of CD154+ cell numbers obtained by ARTE. For each donor, the number of *A. fumigatus*-reactive CD154+ cells (signal) was divided by the number of CD154+ in the unstimulated sample (noise). **(E, F)** *A. fumigatus*-reactive CD154+ T cells were enriched by ARTE, FACS-purified, and expanded in vitro. **(E)** Representative dot plot examples for restimulation with *A. fumigatus* lysate or CMV lysate as control antigen. Flow plots are in part derived from Figure 6A. **(F)** summary of restimulation reactivity of healthy donors (n=11) or pwCF (n=8). **(G)** Stimulation of *A. fumigatus*-reactive CD154+ T cells in presence or absence of an anti-HLA-DR blocking antibody. Cell counts

after CD154+ ARTE enrichment are indicated. **(H)** Enriched cell numbers and percent inhibition of CD154 induction by anti-HLA-DR blockade in different donors (n=11). **(I)** Dot plot examples showing in vitro suppression assay with polyclonal Tregs. Ex vivo isolated Tregs were combined with proliferation dye labeled allogeneic responder CD4+ T cells (Tresp) in different Treg to Tresp ratios. Cells were stimulated polyclonally with CD3/CD28 beads. Numbers indicate percentage of Tresp proliferation. Flow plots are in part derived from Figure 2C. **(J)** Dot plot examples for the in vitro suppression assay with *A. fumigatus*-specific Tregs. Allogeneic CD4+ responder T cells (Tresp) were labeled with cell trace violet dye and stimulated polyclonally with CD3/CD28 beads. Ex vivo isolated *A. fumigatus*-reactive CD137+ Tregs or CD154+ Tcons were added at a T cell: Tresp ratio of 1:4. Numbers indicate percentage of proliferating Tresp cells.
Each symbol in (B, C, D, F, H) represents one donor, horizontal lines indicate geometric mean in (B, C, D); bars and error bars indicate mean and SEM in (F, H). Statistical differences: Two-tailed paired t test in (H).

Supplemental Figure 2

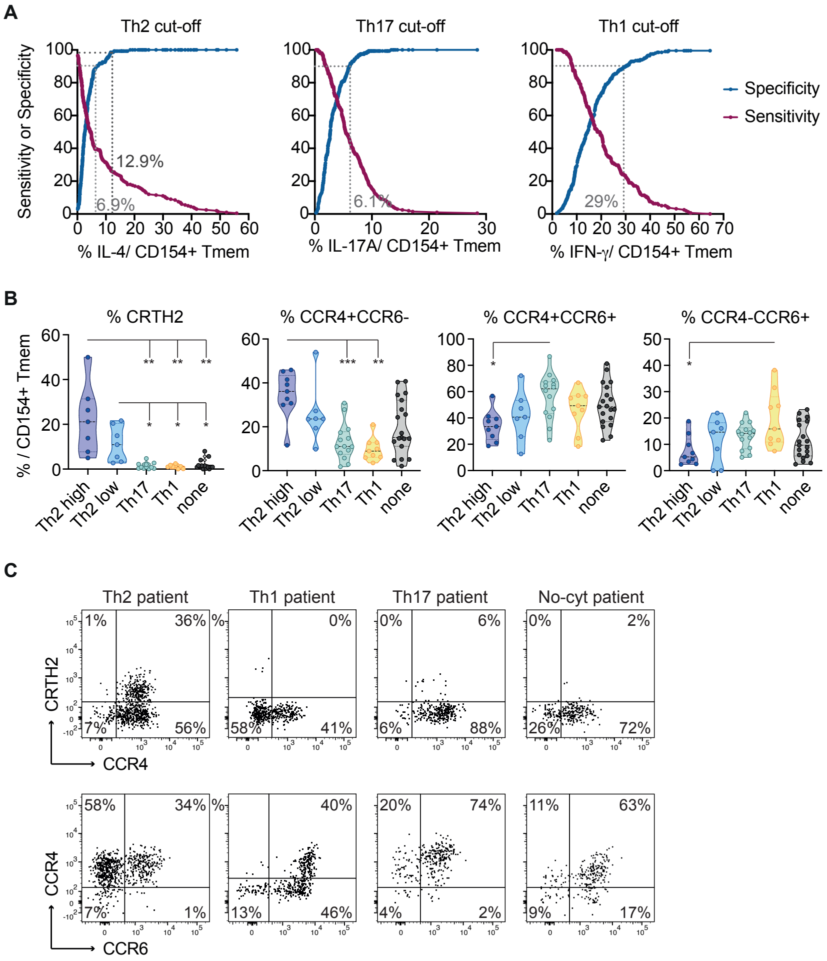

Figure S2. Chemokine receptor pattern of *A. fumigatus*-reactive T cells in pwCF.
(A) Cut-off values for cytokine production of *A. fumigatus*-reactive Tmem to discriminate between healthy individuals and pwCF were calculated by receiver operating characteristic (ROC) analysis and defined to detect patients with 90% specificity. For IL-4 production, a second threshold was set at a sensitivity of 99% to identify patients with particularly high production of IL-4. The derived cut-off values were 6.9% for IL-4-low, 12.9% for IL-4-high, 6.1% for IL-17A, and 29% for IFN-γ producing cells among CD154+ Tmem, respectively. **(B, C)** *A. fumigatus*-reactive T cells from pwCF were stained ex vivo for CRTH2, CCR4 and CCR6 expression. **(A)** Expression within the different *A. fumigatus*-specific T cell reactivity groups is shown. **(B)** Representative dot plot examples. Cells were gated on CD154+ memory cells and percentages of marker positive cells are shown.
Truncated violin plots with quartiles and range are shown in (B), each symbol represents one donor. Statistical differences: Kruskal-Wallis test with Dunn´s post hoc test in (B), significant differences are indicated as ****P<0.0001; ***P<0.001; **P<0.01; *P<0.5.

Supplemental Figure 3

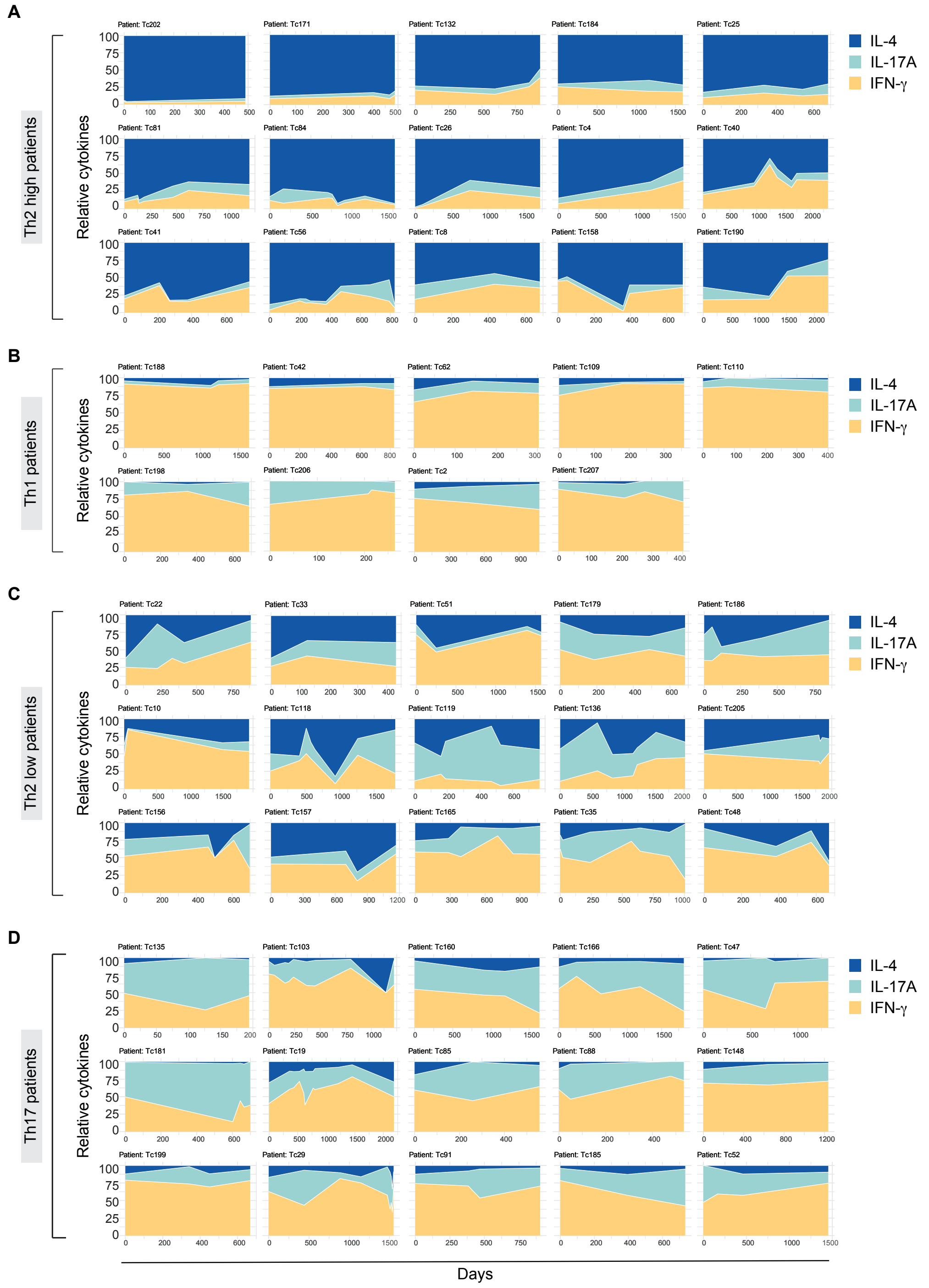

Figure S3. Stability of different anti-*A. fumigatus* T cell reactivity pattern in pwCF.
(A-D) Stability of the different *A. fumigatus*-specific cytokine reactivity patterns. *A. fumigatus*-reactive T cells of pwCF were monitored at least three times over a period of up to 5 years. Relative expression of IFN-γ, IL-17A and IL-4 within CD154+ Tmem is shown for the different reactivity groups: **(A)** Th2 high patients; **(B)** Th1 patients; **(C)** Th2 low patients; **(D)** Th17 patients.

Supplemental Figure 4

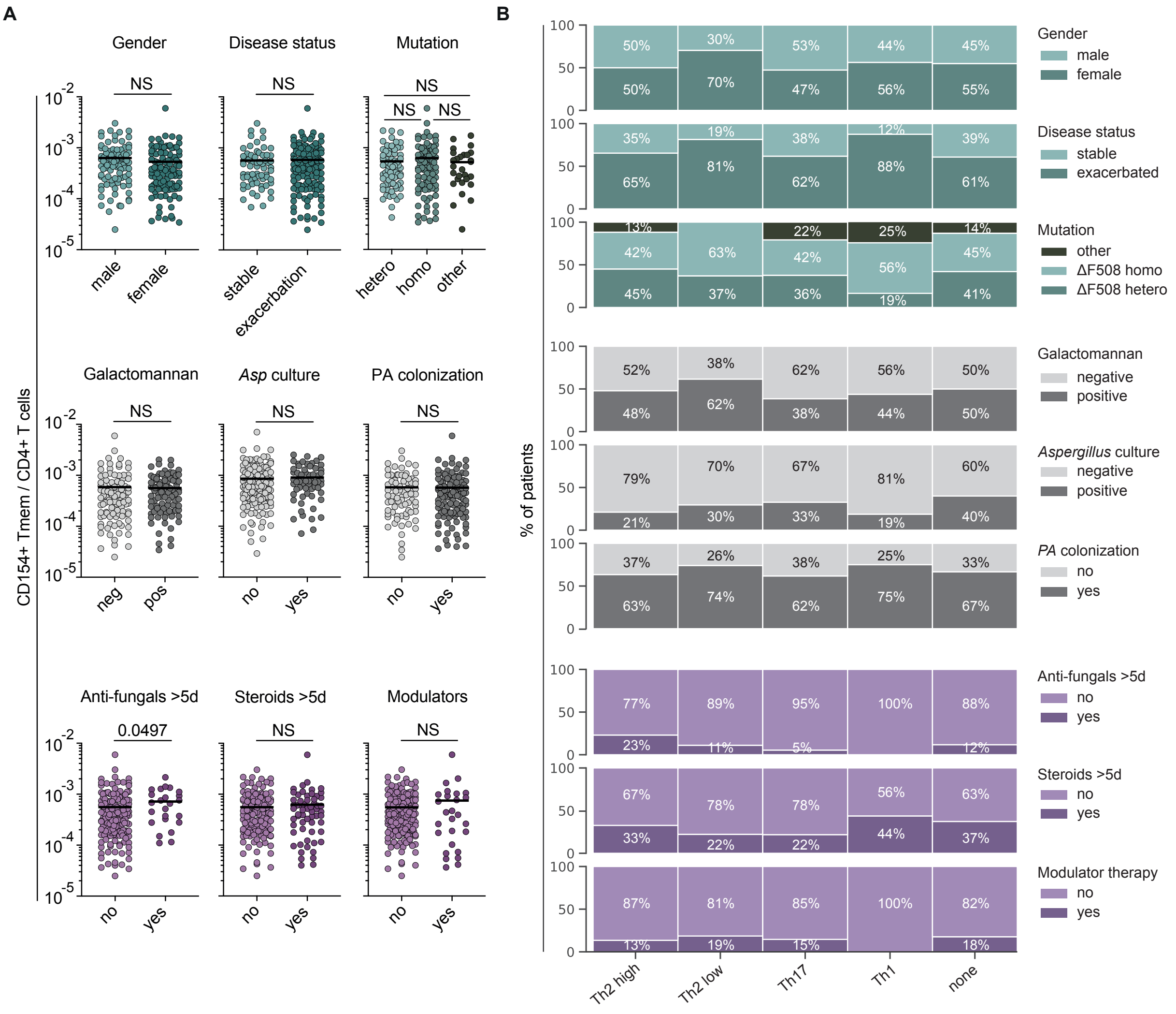

Figure S4. Correlation of *A. fumigatus*-specific T cell data with clinical parameters.
(A) Frequencies of *A. fumigatus*-reactive Tmem are shown for different patient groups.
(B) Distribution of patients according to different clinical parameters within the different T cell reactivity groups. Modulator therapy refers to Ivacaftor single or Ivacaftor/Lumacaftor combination therapy.

Supplemental Figure 5

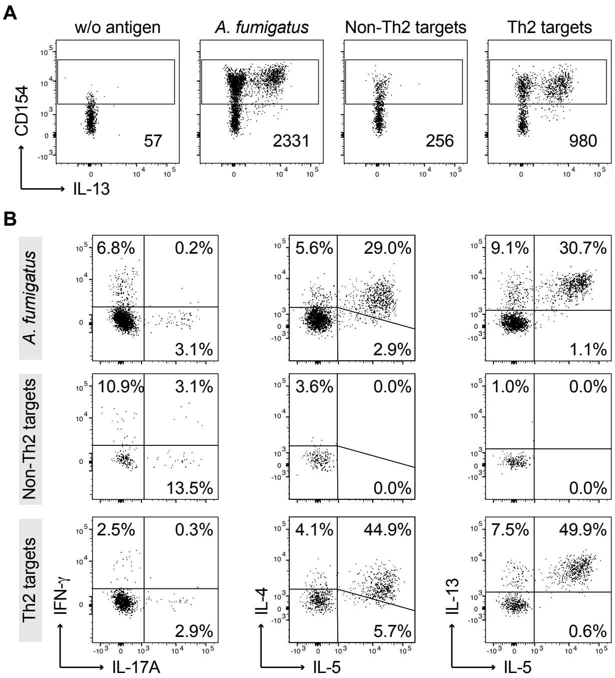

Figure S5. Different target antigens of *A. fumigatus*-reactive T cell subsets.

(A, B) Ex vivo stimulation of a sensitized CF patient with whole *A. fumigatus* lysate or pooled Th2 target proteins (Aspf2, Aspf3, CpcB, CatB, Fg-gap, GliT) and non-Th2 target proteins (Scw4, Aspf22, Pst1, Shm2, CcpA, TpiA, Crf1, Sod3). **(A)** 1×10e7 PBMCs were stimulated or left unstimulated, and cell counts before and after magnetic CD154+ ARTE enrichment are indicated. **(B)** Cells were gated in CD154+ memory cells and percentage of cytokine producers against *A. fumigatus* lysate or the different protein pools are indicated.

Supplemental Figure 6

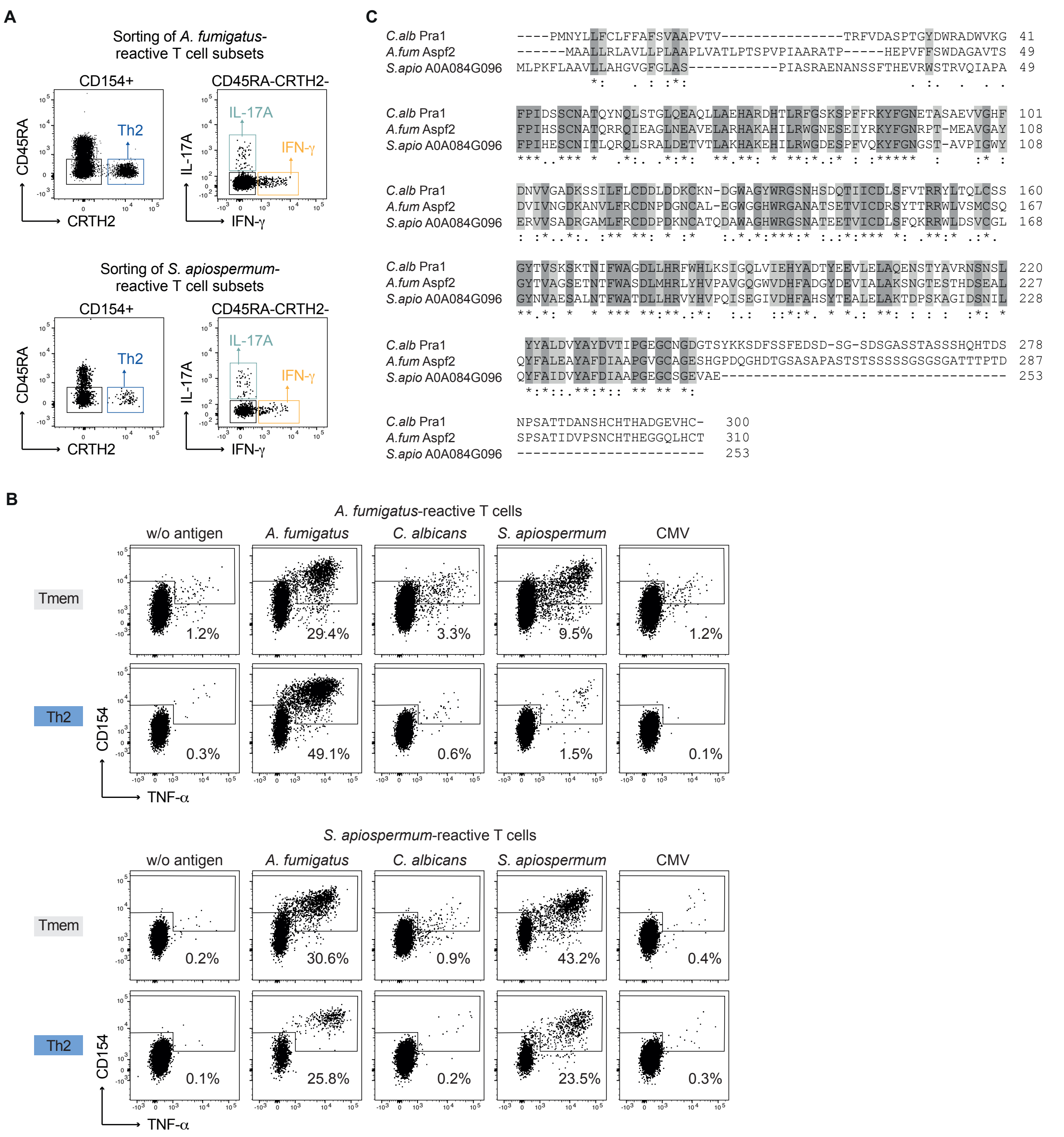

Figure S6. Asymmetric T cell cross-reactivity within *A. fumigatus*- and *S. apiospermum*-activated Th2 cells.

(A) Gating strategy for FACS sorting of *A. fumigatus*- and *S. apiospermum*-reactive T cell subsets from CF patients. Reactive CD154+ cells were magnetically isolated and counterstained for CRTH2 and IL-17A, IFN-γ by combined IL-17A/IFN-γ secretion assay. **(B)** Representative re-stimulation of expanded *A. fumigatus*- and *S. apiospermum* Tmem or Th2 cell lines. Percentage of CD154+TNFα+ within CD4+ is indicated. Flow plots are in part derived from Figure 6A. **(C)** Clustal Omega sequence alignment of the *C. albicans* protein Pra1 (UniProtKB P87020), the *A. fumigatus* protein Aspf2 (UniProtKB P79017) and the *S. apiospermum* protein UniProtKB A0A084G096. Identical and similar amino acids are indicated in dark and light grey, respectively.

Table S1. Cohort characteristics.

	All CF patients (n=201)	CF “Th2-high” group (n=52)	CF “Th2-low” group (n=27)	CF “Th17” group (n=55)	CF “Th1” group (n=16)	CF “none” group (n=51)	Healthy (n=220)
**Age** (mean ± SD, years)	29.6 ± 12.7	27.7 ± 11.0	28.0 ± 13.3	26.7 ± 12.0	29.9 ± 10.4	35.3 ± 13.9	35.9 ± 11.3
**Gender**							
Male (%)	46.3	50.0	29.6	52.7	43.8	45.1	44.7
Female (%)	53.7	50.0	70.4	47.3	56.2	54.9	55.3
**BMI kg/m^2** (mean ± SD)	20.2 ± 3.7	20.1 ± 3.6	19.6 ± 3.2	20.3 ± 4.3	20.7 ± 3.5	20.4 ± 3.7	NA
**CRP (mg/l)** (mean ± SD)	16.4 ± 24.5	15.9 ± 22.9	18.9 ± 20.1	12.7 ± 20.8	17.1 ± 21.7	19.6 ± 32.1	NA
**FEV1 % acute** (mean ± SD)	56.4 ± 27.6	53.1 ± 22.9	55.9 ± 29.4	62.9 ± 25.9	41.0 ± 18.9	58.8 ± 32.8	NA
**FVC %** (mean ± SD)	72.7 ± 24.8	71.6 ± 23.1	71.5 ± 26.3	78.2 ± 22.0	51.8 ± 16.4	75.2 ± 27.8	NA
**Total IgE (kU/l)** (mean ± SD)	299 ± 676	875 ± 1108	163 ± 188	86 ± 176	116 ± 298	72 ± 171	NA
***A.f.* IgE (kU/l)** (mean ± SD)	5.5 ± 12.1	17.5 ± 18.4	4.0 ± 5.9	0.9 ± 2.9	0.2 ± 0.2	0.6 ± 1.2	NA
***A.f.* IgG (mg/l)** (mean ± SD)	67.6 ± 44.3	80.5 ± 47.7	72.2 ± 46.4	61.9 ± 42.0	60.8 ± 41.8	55.2 ± 30.9	NA
**Disease status**							
Stable (%)	32.8	34.6	18.5	38.2	12.5	39.2	NA
Exacerbated (%)	67.2	65.4	81.5	61.8	87.5	60.8	NA
**Mutation**							
∆F508 homozygous (%)	46.8	42.3	63.0	41.8	56.2	45.1	NA
∆F508 heterozygous (%)	38.3	44.2	37.0	36.4	18.8	41.2	NA
Other (%)	14.9	13.5	0.0	21.8	25.0	13.7	NA
**Galactomannan test positive** (%)	47.4	47.9	61.5	38.8	43.8	50.0	NA
**Microbiology**							
Aspergillus culture positive (%)	30.0	21.2	29.6	32.7	18.8	40.0	NA
P. aeruginosa colonization (%)	66.2	63.5	74.1	61.8	75.0	66.7	NA
Nontuberculous Mycobacteria (%)	8.5	13.5	7.4	7.3	6.3	5.9	NA
**Comorbiditis**							
Pancreas insufficiency (%)	89.5	78.9	92.6	92.7	100	92.2	NA
Diabetes (%)	32.8	36.5	33.3	25.5	37.5	35.3	NA
Cystic fibrosis-associated liver disease (CFLD) (%)	9.9	5.8	18.5	9.1	12.5	9.8	NA
Organ Transplantation (%)	5.0	3.9	0	1.8	6.2	11.8	NA
**Medication**							
Anti-fungal treatment >5 days (%)	11.9	23.1	11.1	5.5	0.0	11.8	NA
Steroid treatment >5 days (%)	30.3	32.7	22.2	21.8	43.8	37.3	NA
*Modulator therapy (%)	14.4	13.5	81.5	14.5	0.0	17.6	NA

NA = Not applicable. *Modulator therapy = Ivacaftor single or Ivacaftor/Lumacaftor combination therapy.

Table S2. Results of the sequence similarity searches using NCBI BLAST+ (blastp). *A. fumigatus* protein sequences were compared to the proteome sequence of *S. apiospermum.*

***A. fumigatus* Af293 protein**	Locus-tag	UniProtKB	Length (aa)	***S. apiospermum***	Identifier	UniProtKB	Length (aa)	BLAST Score (bits)	Identity (%)	No. of identical /positive AA
CpcB	AFUA_4G13170	Q4WQK8	316	SAPIO_CDS8541	XP_016640419.1	A0A084FZV8	316	607	91	289/316
Aspf22/EnoA	AFUA_6G06770	Q96X30	438	SAPIO_CDS3461	XP_016644254.1	A0A084GAU3	438	781	87	379/402
Hsp70	AFUA_1G07440	Q4WJ30	638	SAPIO_CDS0158	XP_016646642.1	A0A084GHN1	652	954	85	516/573
HscA	AFUA_8G03930	Q4WCM2	614	SAPIO_CDS5518	XP_016642145.1	A0A084G4T4	614	1057	84	574/614
Afu3g09320 (Shm2)	AFUA_3G09320	Q4WXF4	471	SAPIO_CDS5029	XP_016642937.1	A0A084G726	483	839	84	394/468
Afu5g13450 (TpiA)	AFUA_5G13450	Q4WVV5	256	SAPIO_CDS7740	XP_016641369.1	A0A084K2K8	248	339	65	162/193
Sod3/Aspf6	AFUA_1G14550	Q92450	210	SAPIO_CDS6474	XP_016641856.1	A0A084G3Z5	213	255	62	121/143
CatB/Cat1	AFUA_3G02270	Q92405	728	SAPIO_CDS2912	XP_016644600.1	A0A084GBT9	725	896	61	443/537
Gel1	AFUA_2G01170	P0C7S9	452	SAPIO_CDS8649	XP_016640509.1	A0A084G048	443	478	55	252/317
Aspf2	AFUA_4G09580	P79017	310	SAPIO_CDS8711	XP_016640557.1	A0A084G096	253	260	55	126/160
Aspf3	AFUA_6G02280	O43099	168	SAPIO_CDS9937	XP_016639064.1	A0A084FW03	166	186	54	90/116
Crf1/Aspf9	AFUA_1G16190	Q8J0P4	395	SAPIO_CDS3626	XP_016644386.1	A0A084GB75	371	274	46	145/197
GliT	AFUA_6G09740	E9RAH5	334	SAPIO_CDS10274	XP_016638733.1	A0A084FV22	592	300	45	142/205
CsnB	AFUA_4G01290	Q875I9	238	SAPIO_CDS7894	XP_016640695.1	A0A084G0N4	287	185	45	106/148
Scw4	AFUA_6G12380	Q4WLT6	369	SAPIO_CDS3657	XP_016644415.1	A0A084GBA4	540	134	32	87/128
Pst1	AFUA_6G10290	Q4WMD5	405	SAPIO_CDS7990	XP_016640635.1	A0A084G0H4	198	51	47	25/37
FG-GAP	AFUA_1G04130	Q4WK08	307	SAPIO_CDS1936	XP_016645394.1	A0A084GE33	1297	80	19	42/76
Afu1g13670 (CcpA)	AFUA_1G13670	E9R0F1	260	SAPIO_CDS9039	XP_016639840.1	A0A084FY79	579	37	15	13/28
Aspf1	AFUA_5G02330	P67875	176	-	-	-	-	-	-	-
Aspf4	AFUA_2G03830	O60024	322	-	-	-	-	-	-	-
NadA	AFUA_6G14470	Q4WL81	234	-	-	-	-	-	-	-
ScwA	AFUA_4G09310	Q4WPH8	183	-	-	-	-	-	-	-

Grey letters indicate no significant similarity and "-" indicates that no orthologue proteins were identified.
